# Anticachectic regulator analysis reveals Perp-dependent antitumorigenic properties of 3-methyladenine in pancreatic cancer

**DOI:** 10.1172/jci.insight.153842

**Published:** 2022-01-25

**Authors:** Aneesha Dasgupta, Paige C. Arneson-Wissink, Rebecca E. Schmitt, Dong Seong Cho, Alexandra M. Ducharme, Tara L. Hogenson, Eugene W. Krueger, William R. Bamlet, Lizhi Zhang, Gina L. Razidlo, Martin E. Fernandez-Zapico, Jason D. Doles

**Affiliations:** 1 Department of Biochemistry and Molecular Biology,; 2 Schulze Center for Novel Therapeutics, Division of Oncology Research, Department of Oncology,; 3 Division of Gastroenterology and Hepatology,; 4 Health Sciences Research, and; 5 Department of Laboratory Medicine and Pathology, Mayo Clinic, Rochester, Minnesota, USA.

**Keywords:** Muscle Biology, Oncology, Cancer, Expression profiling, Skeletal muscle

## Abstract

Approximately 80% of pancreatic cancer patients suffer from cachexia, and one-third die due to cachexia-related complications such as respiratory failure and cardiac arrest. Although there has been considerable research into cachexia mechanisms and interventions, there are, to date, no FDA-approved therapies. A major contributing factor for the lack of therapy options could be the failure of animal models to accurately recapitulate the human condition. In this study, we generated an aged model of pancreatic cancer cachexia to compare cachexia progression in young versus aged tumor-bearing mice. Comparative skeletal muscle transcriptome analyses identified 3-methyladenine (3-MA) as a candidate antiwasting compound. In vitro analyses confirmed antiwasting capacity, while in vivo analysis revealed potent antitumor effects. Transcriptome analyses of 3-MA–treated tumor cells implicated *Perp* as a 3-MA target gene. We subsequently (a) observed significantly higher expression of *Perp* in cancer cell lines compared with control cells, (b) noted a survival disadvantage associated with elevated *Perp*, and (c) found that 3-MA–associated *Perp* reduction inhibited tumor cell growth. Finally, we have provided in vivo evidence that survival benefits conferred by 3-MA administration are independent of its effect on tumor progression. Taken together, we report a mechanism linking 3-MA to *Perp* inhibition, and we further implicate *Perp* as a tumor-promoting factor in pancreatic cancer.

## Graphical abstract

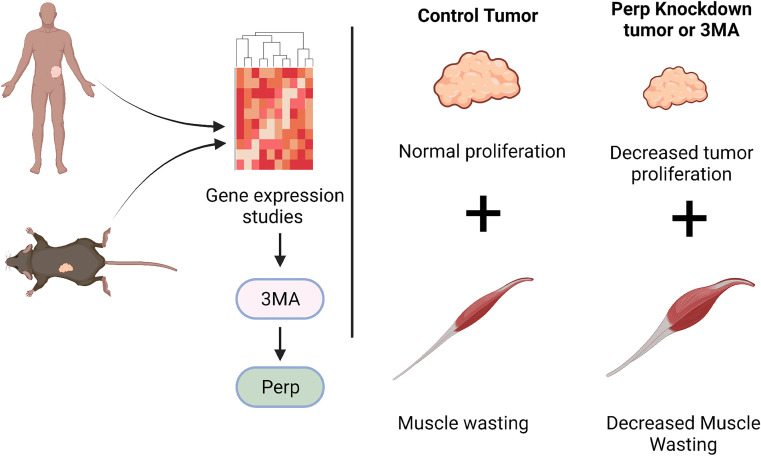

## Introduction

Pancreatic cancer is the third leading cause of cancer-related deaths in the United States. By 2030, it will be the second leading cause (1, 2). The low 5-year survival of ~10% is largely due to late diagnosis, early metastasis, resistance to conventional therapy, and pervasive cachexia, or muscle wasting. Although cachexia is not often listed as a primary cause of death, it is widely understood that patients experiencing weight and muscle loss have worse prognoses than those who do not (3, 4). Furthermore, many cachectic cancer patients either fail to qualify for chemotherapy or are refractory to pharmacological intervention. Although significant progress has been made toward identifying fundamental mechanisms of cancer cachexia, FDA-approved therapies are lacking (5). Major reasons for the lack of effective therapies are likely rooted in fundamental differences between animal models and the human condition (6). One variable likely contributing to failure at the clinical trial stage is inadequate consideration of physiological age in animal models. Most preclinical studies utilize mice 6 to 8 weeks of age — roughly corresponding to a human age of 20 years — despite overwhelming evidence documenting substantial metabolic, physiological, and molecular differences in young versus aged skeletal muscle (7–9). Given that the median age of diagnosis of pancreatic cancer is 70, it reasons that therapeutic targets identified based on molecular changes observed in young mouse models of cancer cachexia might not translate effectively to an aged human population.

In the present study, we queried transcriptional changes in the skeletal muscle of young and aged mice orthotopically implanted with pancreatic cancer cells. Based on changes observed in aged wasting muscle, we identified 3-methyladenine (3-MA) as a candidate antiwasting compound. 3-MA is well known as an inhibitor of phosphatidylinositol 3-kinases (PI3Ks) and modulator of autophagy. The precise effect of 3-MA on autophagy is complex, in part due to effects on multiple PI3K isoforms (10). Classically, 3-MA is reported to inhibit autophagy by blocking autophagosome formation via class III PI3K inhibition (10). Prolonged treatment, however, can promote autophagy under nutrient-rich conditions or inhibit starvation-induced autophagy — an effect hypothesized to involve both class I and class III PI3K inhibition (10). In disease intervention contexts, 3-MA can (a) inhibit atherosclerotic lesion progression (11), (b) protect against apoptosis in a model of subarachnoid hemorrhage (12), (c) improve endothelial/barrier cell dysfunction in acute lung injury (13), and (d) improve survival in endotoxemia and polymicrobial sepsis models (14). With respect to cancer, 3-MA can increase therapeutic efficacy (15), as well as inhibit cell migration and invasion (16). Both autophagy-dependent and -independent cell death pathways are reported to mediate the effects of 3-MA in cancer cells (17). Comprehensive studies linking 3-MA to inhibition of pancreatic cancer progression and cancer cachexia are lacking.

Our data implicate PERP (P53 Apoptosis Effector Related to PMP22), a tetraspan protein localized in the plasma membrane, as a potentially novel 3-MA target. *Perp* is best known as a p53 target gene (18) preferentially involved in apoptosis, as opposed to cell cycle arrest. This assertion arose in large part due to observations that *Perp* is more highly expressed in cells undergoing p53-dependent apoptosis compared with those undergoing p53-dependent G1 arrest (18). Subsequent studies expanded PERP function to include an essential role in adhesion and epithelial integrity (19). There are conflicting reports as to whether *Perp* promotes or suppresses tumorigenesis. In a recent study, METTL14-mediated *Perp* reduction led to increased tumor cell proliferation and metastasis (20). There are, however, reports that *Perp* deletion antagonizes oncogenic progression (21). In this study, we provide evidence that *Perp* potentiates tumor cell growth. Overall, we (a) highlight the utility of age-appropriate cancer models to identify novel cachexia-associated pathways/targets, (b) identify 3-MA as dual inhibitor of cancer-associated muscle atrophy and pancreatic tumor progression, and (c) implicate *Perp* as a potentially novel 3-MA target and pancreatic cancer oncogene.

## Results

### KPC-derived cancer cells promote cachexia upon orthotopic transplantation into young and aged mice.

**Figure 1:**
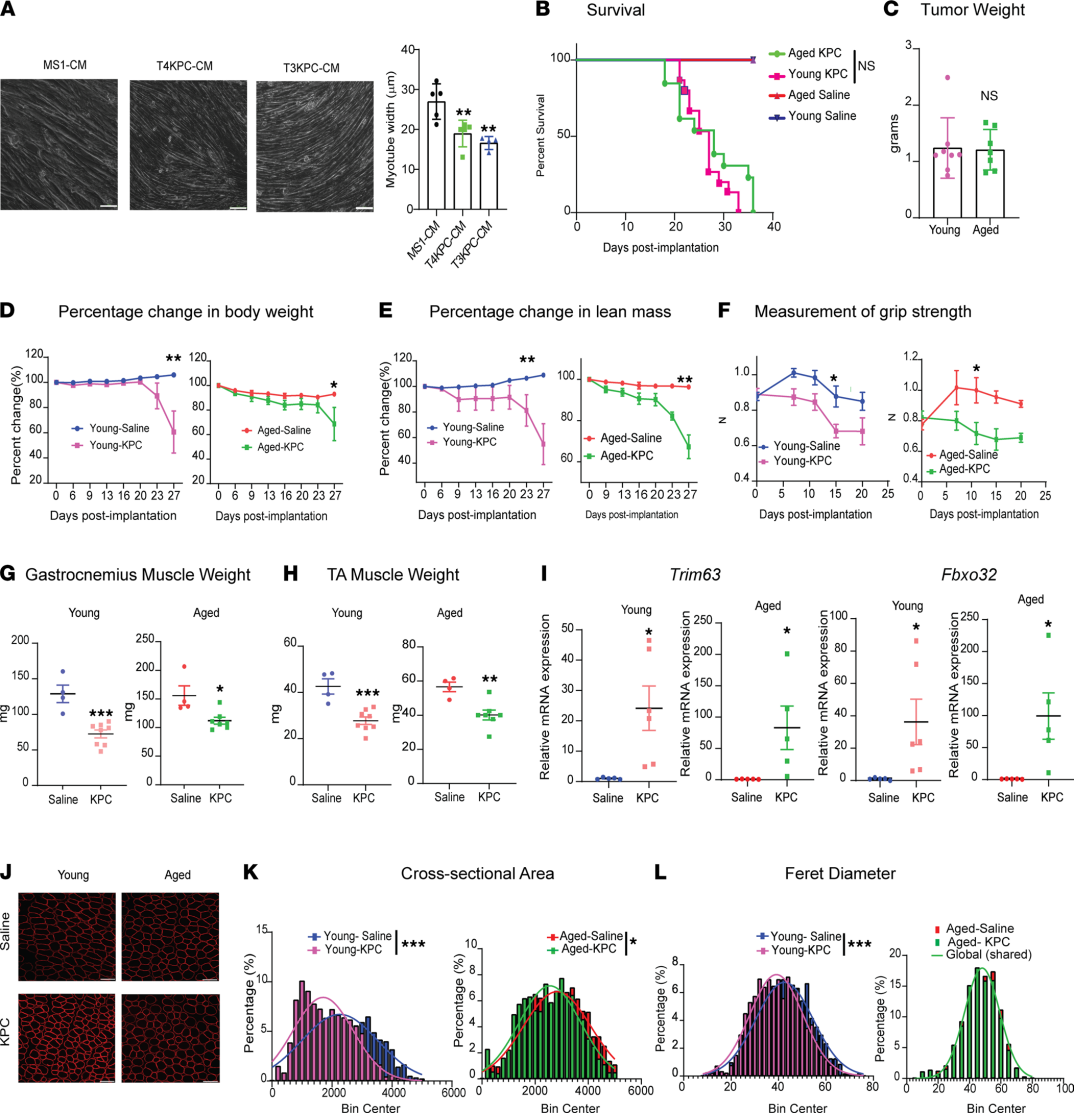
KPC cells promote in vitro myotube atrophy and in vivo muscle wasting. (**A**) (Left) Representative micrograph images (200×) of differentiated C2C12 myotubes treated with MS1 CM, T4-KPC CM, and T3-KPC CM for 24 hours. The experiment was conducted at least 3 times .(Right) Quantification of myotube width. Scale bar: 100 μm. (**B**) Kaplan-Meier survival curve for young and aged mice; saline-injected (*n* = 5 each) and T4-KPC cell–injected (*n* = 15 for young and *n* = 13 for aged). (**C**) Postnecropsy quantification of tumor weight from young and aged tumor-bearing mice (*n* = 8 for young KPC; *n* = 7 for aged KPC). (**D**–**F**) Longitudinal quantification of body weight, lean mass, and grip strength. (**G** and **H**) Postnecropsy measurement of gastrocnemius and tibialis anterior wet weights in young/aged control and tumor-bearing mice (*n* = 4 for saline controls, *n* = 8 for young KPC, *n* = 7 for aged KPC). (**I**) mRNA expression of *Trim63* and *Fbxo32* in the gastrocnemius muscles of young/aged control and tumor-bearing mice (*n* = 5 in each group). (**J**) Laminin staining of fixed gastrocnemius tissue cross-sections. Scale bar: 100 μM. (**K** and **L**) Quantification of cross-sectional area and minimum feret diameter of the laminin-stained gastrocnemius tissue sections. Minimum feret diameters were binned to a histogram and fit with a nonlinear regression (Gaussian, least-squares regression). Data are mean ± SEM, compared with 1-way ANOVA with Bonferroni’s (**A**), Log-rank test (Mantel Cox) (**B**), 2-tailed Student’s *t* test (**C** and **G**–**I**), and 2-way ANOVA with Bonferroni’s (**D**–**F**). *P < 0.05; **P < 0.01; ***P < 0.001.

We first aimed to query the cachexia-inducing properties of T4- and T3-KPC (*Kras^LSL.G12D/+^*; *p53^LSL.R172H/+^*; *Pdx1-Cre*) pancreatic cancer cells in vitro. An established conditioned media (CM)/C2C12 myotube atrophy model was used (22, 23). We observed a reduction in mean myotube diameter of C2C12 cells treated with T4-KPC CM and T3-KPC CM (*P* = 0.0069 and 0.0018, respectively) compared with those treated with MS1 (a noncancerous pancreas endothelial cell line) CM (Figure 1A). On a molecular level, we observed induction of the muscle-specific ubiquitin ligases MuRF1/*Trim63* and Atrogin-1/*Fbxo32*, as well as a reduction in myosin heavy chain expression (Supplemental Figure 1, A and B; supplemental material available online with this article; https://doi.org/10.1172/jci.insight.153842DS1). Next, we orthotopically implanted T4-KPC cells into the pancreas of differently aged recipient mice to query the effect of host age on cachexia/tumor progression. We observed no significant changes in overall survival, longitudinal tumor growth, or terminal tumor weight between the 2 cohorts (Figure 1, B and C, and Supplemental Figure 1C). Longitudinal measurements revealed significant decreases in overall body weight, lean mass, and grip strength, as well as a nonsignificant decrease in fat mass in tumor-bearing mice compared with saline-injected control mice (Figure 1, D–F, and Supplemental Figure 1D). We also observed increases in several wasting-associated cytokines (i.e., IL-6 and TNF-α) in the serum of young and aged tumor-bearing mice, suggesting that systemic inflammation is a common feature of both models (Supplemental Figure 1E).

Postnecropsy measurements revealed reductions in gastrocnemius (young [8 weeks], *P* = 0.0006; aged [78 weeks], *P* = 0.0158) and tibialis anterior (TA) muscle mass (young, *P* = 0.001; aged, *P* = 0.004) in tumor-bearing mice compared with controls (Figure 1, G and H). Consistent with in vitro observations, *Trim63* (young, *P* = 0.0187; aged, *P* = 0.045) and *Fbxo32* (young, *P* = 0.0491; aged, *P* = 0.0262) gene expression was increased in skeletal muscle lysates prepared from tumor-bearing mice (Figure 1I). Laminin immunostaining and subsequent assessment of myofiber cross-sectional area (CSA) revealed a significant decrease in CSA in tumor-bearing mice in both age cohorts (young, *P* = 0.0003; aged, *P* = 0.03). In contrast, minimum feret diameter measurements were significantly decreased exclusively in the young cohort (*P* < 0.0001) (Figure 1, J–L). Qualitative visual inspection of tissue cross-sections revealed extensive myofiber rounding in aged tumor-bearing muscle, a phenotype absent in control and young tumor-bearing samples. While not widely reported in murine cachexia models, this phenomenon is frequently observed in muscle biopsies from pancreatic cancer patients (22). Taken together, we confirm that the KPC pancreatic cancer cells used in this study have the potential to induce cachexia in both young and aged recipient mice. While morphological and histopathological readouts are comparable, subtle differences exist that suggest that aged mice may more precisely recapitulate the human condition.

### Transcriptomic analyses identify 3-MA as a candidate mediator of muscle wasting.

We next aimed to define the molecular mechanism(s) associated with muscle wasting in young versus aged KPC mice. RNA-Seq analysis of the gastrocnemius muscle was performed on control and tumor-bearing young and aged mice (4 experimental groups). Substantial transcriptomic differences were observed between aged and young control skeletal muscle samples (Supplemental Figure 2, A and B). Seventy-seven differentially expressed genes (DEGs) were identified and included transcripts previously linked to normal skeletal muscle aging and/or function including: *Actc1*, *Col1a1*, *Col1a2*, *Col3a1*, *Ighg2c*, *Igkc*, and *Sln* (Supplemental Figure 2C). Since these genes encode for proteins critical to muscle identity and function (actin α cardiac muscle, collagen, immunoglobulin, sarcolipin, etc.), this observation underscores the significance of age-associated differences in peripheral tissue gene expression and highlights an opportunity to identify and study novel genes/pathways associated with cancer cachexia in a more relevant physiological context.

**Figure 2:**
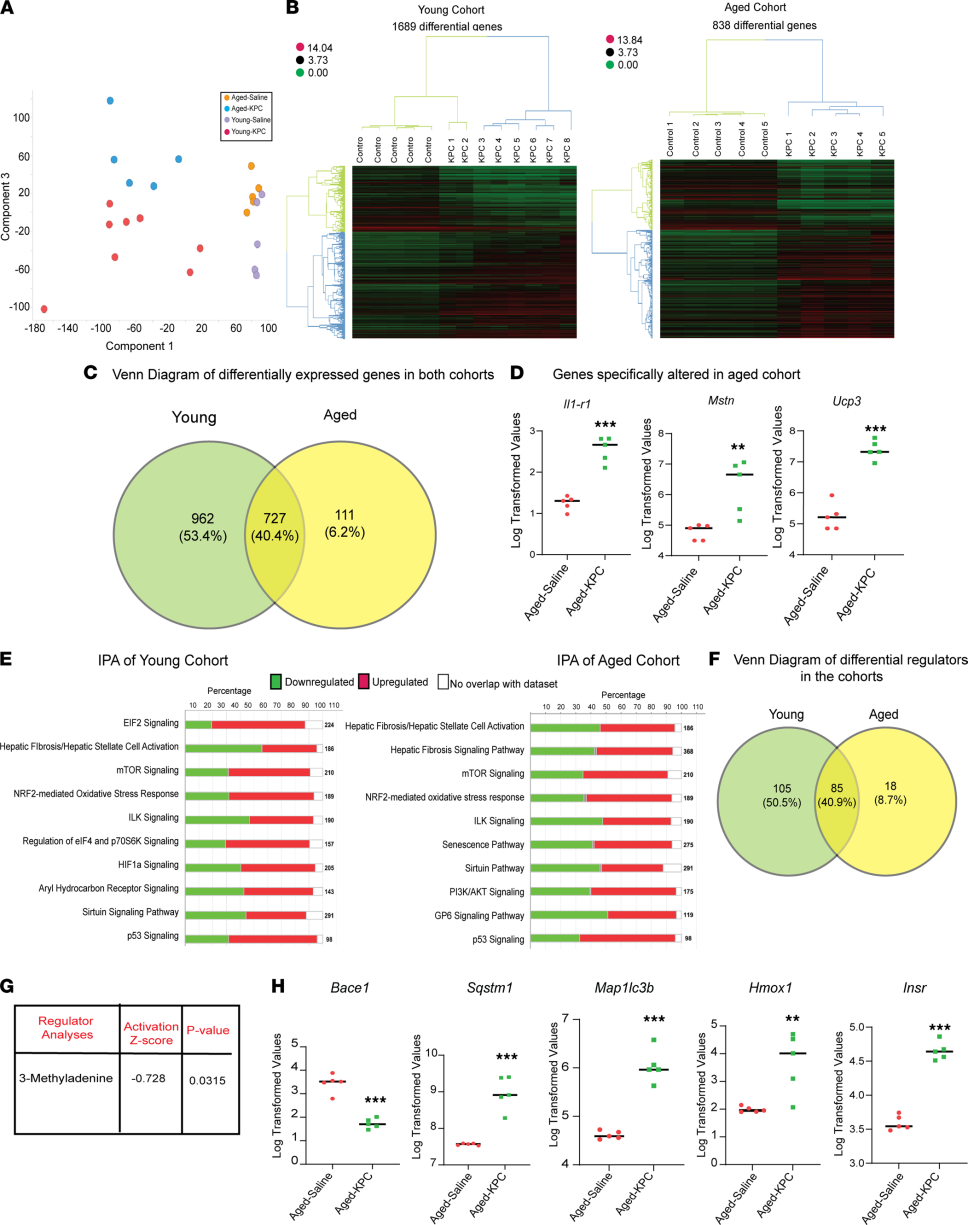
Comparative transcriptome analyses of young and aged skeletal muscle from control and tumor-bearing mice. (**A**) Principal component analysis (PCA) plot depicting global differences in the muscle transcriptome of young/aged control and tumor-bearing mice (*n* = 5 for aged/young controls and aged KPC, *n* = 8 for young KPC). (**B**) Heatmaps depicting differentially expressed genes (DEGs; log-transformed and row-normalized) between the control and tumor-bearing groups in the young (left) and aged (right) cohorts. (**C**) A Venn diagram depicting distinct and common DEGs between young and aged cohorts. (**D**) Log-transformed FPKM values of genes specifically altered in the aged cohort — IL-1 receptor type 1 (*Il1-r1*), Myostatin (*Mstn*), Uncoupling Protein 3 (*Ucp3*). (**E**) Ingenuity Pathway Analyses (IPA) of young (left) and aged (right) DEGs. (**F**) A Venn diagram depicting the distinct and common compounds predicted to reverse the cachectic phenotype in the young and aged cohorts. (**G**) Activation *Z* score and *P* value of 3-Methyladenine (3-MA), a top candidate compound identified based on aged control/KPC DEGs. (**H**) Log-transformed FPKM values of genes that contributed to the 3-MA prediction: β secretase 1 (*Bace1*), Sequestosome 1 (*SQSTM1*), Microtubule Associated Protein 1 Light Chain 3 β (*MAP1LC3B*), Heme Oxygenase 1 (*Hmox1*), Insulin receptor (*INSR*). Data are mean ± SEM, compared with 2-tailed Student’s *t* test (**D** and **H**). **P < 0.01; ***P < 0.001.

Principal component (Figure 2A) and hierarchical clustering (Figure 2B) analyses of transcriptome data from young control/KPC and aged control/KPC cohorts revealed further age-associated group separation. Differential gene expression analyses (based on a cut-off of *P* < 0.05, adjusted *P* < 0.1, and fold change of 2) identified 1689 DEGs in young control versus KPC, while ~50% fewer DEGs (838 DEGs) were identified in aged control/KPC comparisons. This observation was consistent with a recent study demonstrating fewer DEGs in slower progressing cachexia models, as well as in human data sets (24). A total of 727 of these DEGs was shared between young and aged cohorts, while 111 DEGs were exclusively altered in aged KPC muscle (Figure 2C). Of note, we observed upregulation of *Il1-r1*, *Mstn*, and *Ucp3* exclusively in the aged cohort. These transcripts are associated with cancer-associated muscle wasting in humans (25–27) but, like myofiber rounding, are not typically linked to murine cancer–associated muscle wasting (Figure 2D). We next performed validated pathway and regulator analyses (28–31) and prioritized several candidate compounds predicted to reverse the aged muscle-specific atrophy signature (Figure 2E). Eighteen compounds were exclusively identified in the aged data set (Figure 2F). 3-MA emerged as a top candidate-of-interest (*P* = 0.0315) based on (a) a documented ability to rescue muscle wasting in chronic kidney disease models (32, 33), (b) no previous association with pancreatic cancer cachexia, (c) its role as a metabolite with no previous demonstration of substantial in vivo toxicity, and (d) its association with autophagy regulation. Indeed, multiple autophagy (i.e., *Sqstm1*, *Map1lc3b*) and PI3K target (i.e., *Insr*, *Hmox*) genes contributed to the 3-MA prediction in wasting muscle (Figure 2, G and H).

### 3-MA prevents cancer-associated lean mass loss and decreases tumor growth.

**Figure 3:**
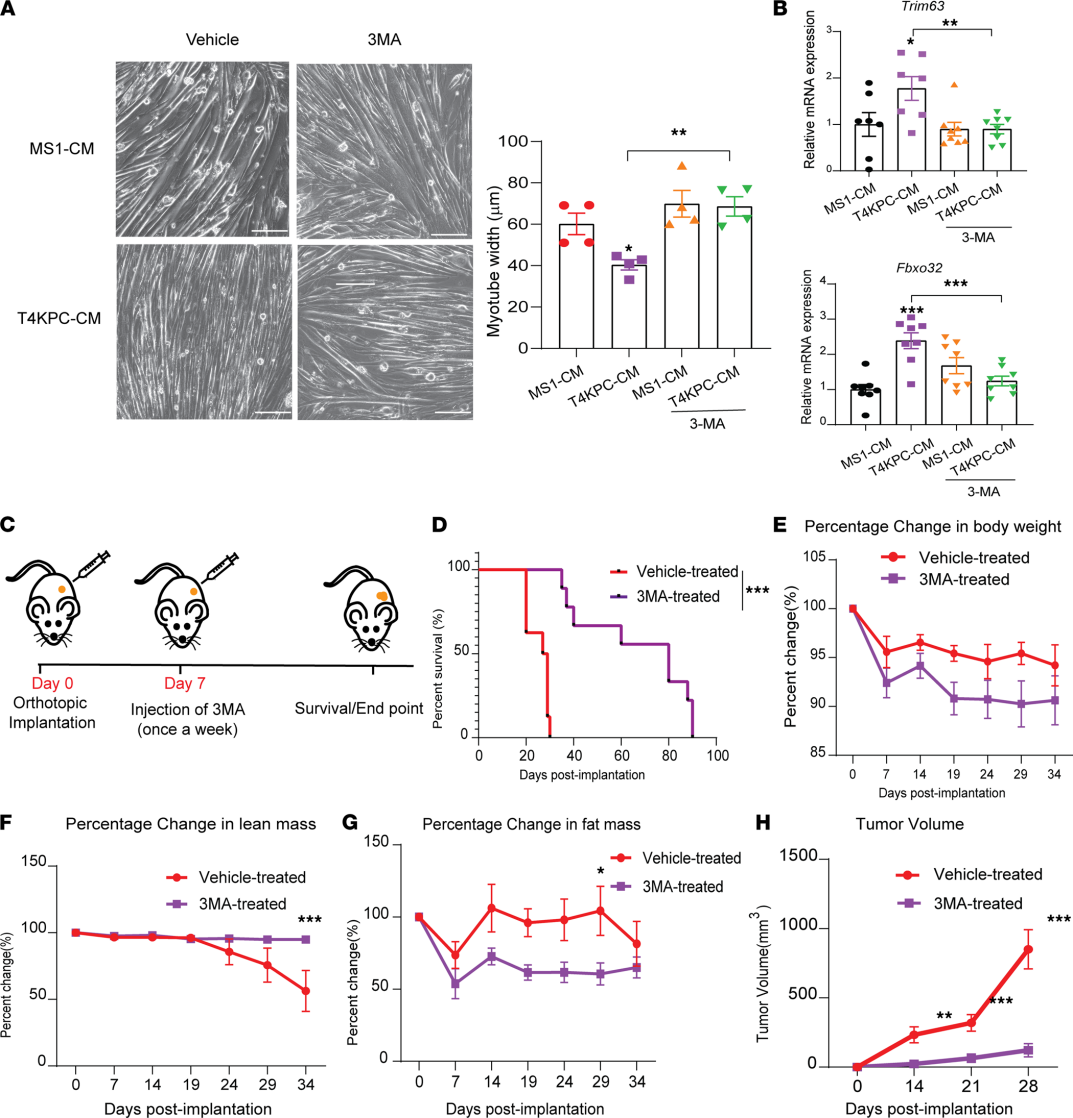
3-MA prevents myotube atrophy, limits cancer-associated lean mass loss, and antagonizes tumor growth. (**A**) (Left) Representative micrograph images (20×) of C2C12 myotubes treated with MS1 and T4-KPC CM with and without 3-MA. (Right) Quantification of myotube diameters. Experiment was repeated ≥ 3 times. Scale bar: 100 μm. (**B**) mRNA expression of *Trim63* and *Fbxo32* in the C2C12 myotubes treated with MS1 and T4-KPC CM with and without 3-MA (10 μM). (**C**) Schematic illustration of tumor implantation and treatment schedule. (**D**) Survival analyses of tumor-bearing mice (aged, 78 weeks) treated with vehicle or 3-MA (*n* = 10 each). (**E**–**G**) Longitudinal measurement of body weight, lean mass, fat mass, and tumor volume of vehicle- and 3-MA–treated mice. Data are mean ± SEM, compared with 1-way ANOVA with Bonferroni’s (**A** and **B**), log-rank test (Mantel-cox) (**D**), and 2-way ANOVA with Bonferroni’s (**E**–**H**). *P < 0.05; **P < 0.01; ***P < 0.001.

As a first step toward determining the potential therapeutic benefit of 3-MA in the context of cancer cachexia, we asked if 3-MA could prevent myotube atrophy induced by CM collected from KPC cells. Concurrent treatment of myotubes with T4-KPC CM and 3-MA was able to prevent CM-associated atrophy (*P* = 0.031) (Figure 3A) and suppress expression of the atrophy transcripts *Trim63* (MuRF1) and *Fbxo32* (Atrogin-1) (*P* = 0.0072 and 0.0004, respectively) (Figure 3B). CM from MS1 cells had no measurable effect on myotube diameter or atrophy marker expression, and 3-MA did not further alter these experimental variables, suggesting that 3-MA is not simply inducing hypertrophy in T4-KPC cultures but, rather, directly mitigating CM-associated atrophy.

We next sought to determine if in vivo 3-MA administration could attenuate muscle wasting in aged KPC mice. T4-KPC cells were orthotopically transplanted into the pancreas of aged mice and treated with vehicle or 3-MA (Figure 3C). We observed a significant survival advantage of the 3-MA–treated cohort (Figure 3D). There was no significant difference in body weight between the 2 groups, but we did observe significant preservation of lean mass upon 3-MA administration (Figure 3, E and F). Fat mass in 3-MA–treated mice trended lower in the 3-MA cohort compared with controls, although this difference was not consistently statistically significant (Figure 3G). Strikingly, we observed a sharp decrease in tumor volume in tumor-bearing mice treated with 3-MA (Figure 3H).

Recognizing the limitations associated with using a single cancer model, we aimed to corroborate these 3-MA effects using an independent, human-relevant, in vivo pancreatic cancer model. To that end, we evaluated the potential of implanting patient-derived organoids (PDOs) into NOD recipient mice to model pancreatic cancer. First, we confirmed that implanted organoids generate pancreas tumors with histopathological features reminiscent of those observed in patients (Supplemental Figure 3, A–C). Next, we asked if 3-MA could augment tumor growth and muscle wasting in this PDO tumor model (Supplemental Figure 3D). While we observed no significant change in body weight between experimental groups, lean mass was significantly elevated and tumor burden was reduced in 3-MA/PDO mice compared with vehicle/PDO control mice (Supplemental Figure 3, E–G). Moreover, 3-MA/PDO mice had a significant survival advantage over control mice (Supplemental Figure 3H). Consistent with the KPC study, we further observed that 3-MA adversely affected fat mass (Supplemental Figure 3I). Together, these observations confirmed that 3-MA was capable of antagonizing tumor growth (and preserving lean mass) using tumor cells of both mouse and human origin, thus underscoring the translational potential of this therapeutic approach.

**Figure 4:**
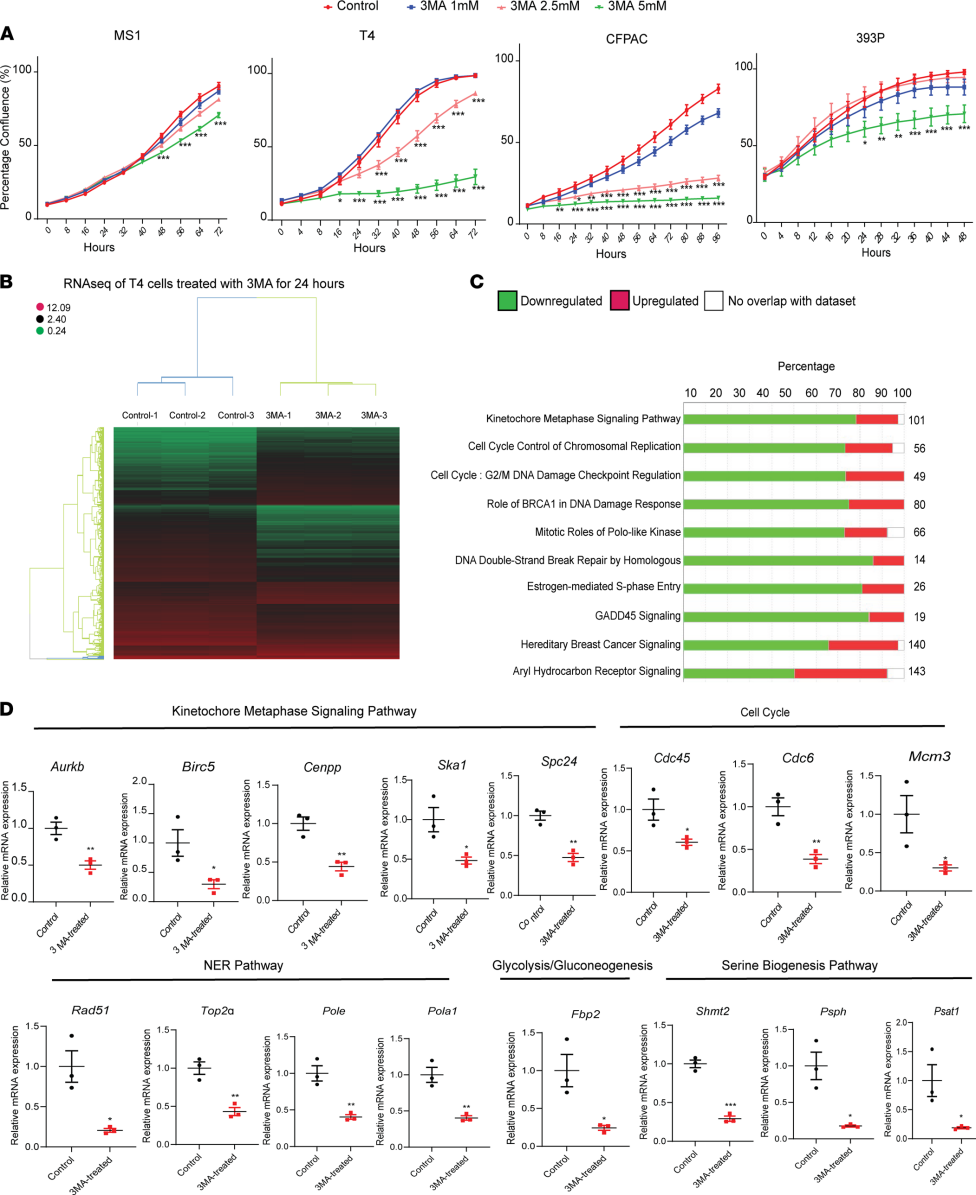
Comparative analyses of tumor cell lines treated with 3-MA. (**A**) Proliferation curves depicting MS1, T4, CFPAC, and 393P cells treated with increasing doses of 3-MA. Experiment was repeated ≥ 3 times. (**B**) A heatmap of differentially expressed genes (log-transformed and row normalized) in T4-KPC cells treated with vehicle or 5 mM 3-MA for 24 hours (*n* = 3). (**C**) Ingenuity pathway analysis (IPA) of 3-MA responsive DEGs in T4-KPC cells. (**D**) Graphs depicting quantitative PCR validation of mRNA expression of selected genes (representing the top IPA pathways) in T4-KPC cells with or without 3-MA. Data are mean ± SEM, compared with 2-tailed Student’s *t* test. *P < 0.05; **P < 0.01; ***P < 0.001.

In light of observations documenting antitumor effects of 3-MA using multiple in vivo models, our next objective was to study this tumor-suppressive effect in greater detail. First, we performed dose-response experiments using MS1, T4-KPC, CFPAC, and 393P cells. MS1, the noncancerous cell line of pancreatic origin, only demonstrated slight cytotoxic effects/sensitivity to 3-MA at the highest dose. Conversely, pancreatic cancer cell lines of mouse (T4-KPC) and human (CFPAC) origin exhibited dose-dependent decreases in cellular proliferation. Notably, 393P lung adenocarcinoma cells (similarly harboring *Kras* and *p53* mutations) were not as sensitive to 3-MA as cancer cells of pancreatic origin and only exhibited proliferation attenuation at the highest 3-MA dose tested (Figure 4A).

To gain molecular insights into mechanisms responsible for 3-MA–mediated tumor cell cytotoxicity, we performed RNA-Seq on cultured T4-KPC cells with or without 3-MA (5 mM). Hierarchical clustering analysis revealed significant separation of control and 3-MA–treated samples (Figure 4B). While 3-MA is reported to inhibit autophagy by acting on the Class III PI3-kinase VPS34 (34), we did not observe notable enrichment of transcripts involved in PI3K/autophagy signaling. Instead, pathway analyses implicated alterations in kinetochore metaphase signaling, cell cycle, mitotic, and DNA damage pathways in response to 3-MA (Figure 4C). Selected downregulated genes in kinetochore metaphase signaling, cell cycle, nucleotide excision repair, glycolysis/gluconeogenesis, and serine biogenesis pathways were validated by quantitative PCR (qPCR), confirming the broad impact of 3-MA treatment on cancer cells (Figure 4D). Additionally, gene expression analyses of endpoint tumor samples collected from control and 3-MA–treated tumor-bearing mice corroborated these in vitro data, thus mitigating potential concerns associated with cell culture expression artifacts (Supplemental Figure 4).

### 3-MA decreases tumor proliferation via Perp inhibition.

**Figure 5:**
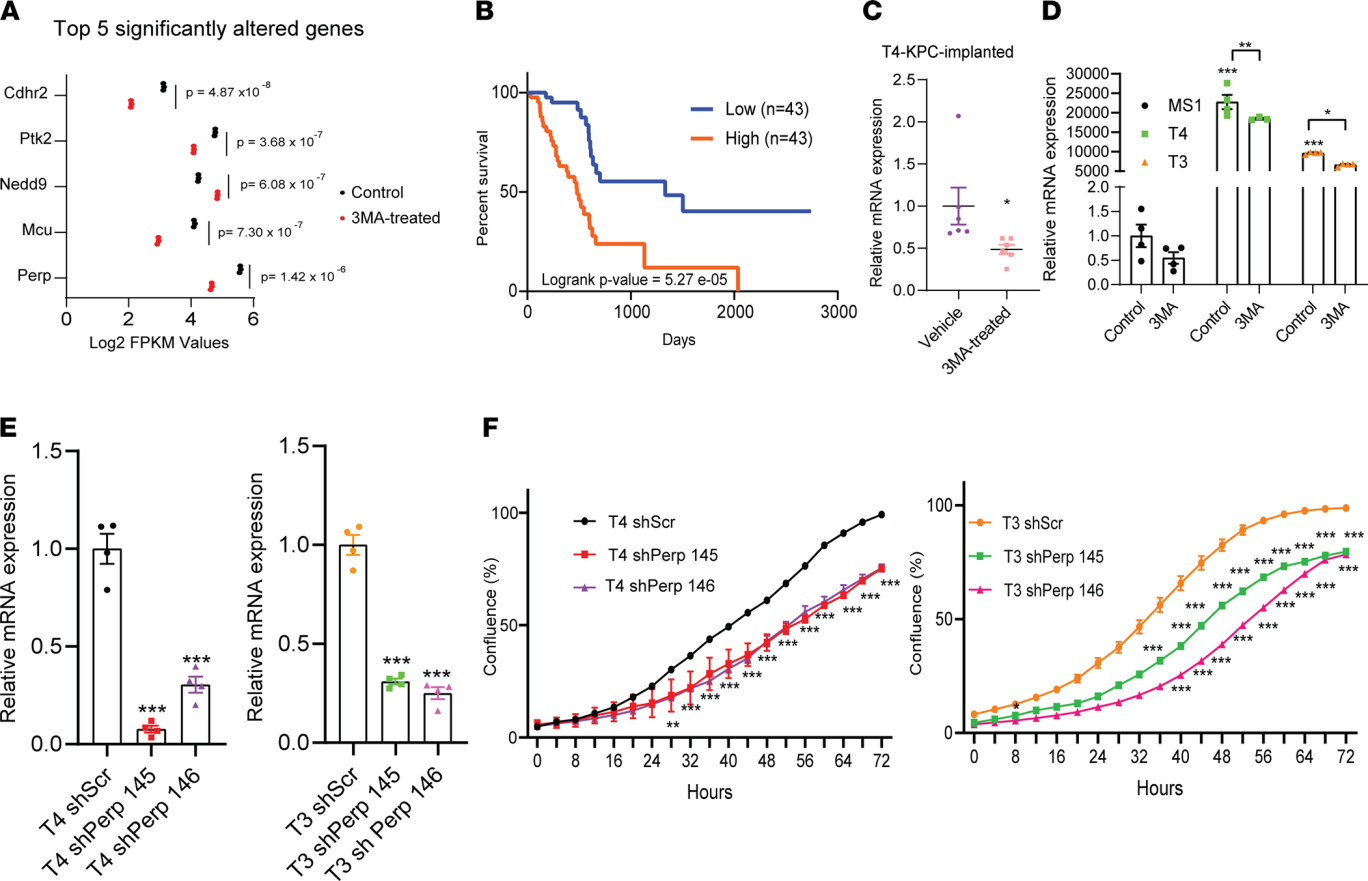
*Perp* is elevated in tumor cells and is antagonized by 3-MA. (**A**) A graph depicting the top 5 DEGs (T4-KPC cells with or without 3-MA) ordered by *P* value. (**B**) Survival analyses of patients (PDAC) having low and high *Perp* expression in the TCGA database. (**C**) In vivo *Perp* mRNA expression in T4-KPC cells with or without 3-MA (*n* = 6 in each group). (**D**) *Perp* mRNA expression in MS1, T4-KPC, and T3-KPC cells with or without 3-MA. (**E**) *Perp* mRNA expression in T4-KPC and T3-KPC (shSCR, Perp shRNA 145, and Perp shRNA 146). (**F**) Line graphs depicting cellular proliferation of T4 shScr/Perp shRNA 145/Perp shRNA 146 (left) and T3 shScr/Perp shRNA 145/Perp shRNA 146 (right) cells. Experiment was repeated ≥ 3 times. Data are mean ± SEM compared with log-rank test (**B**), 2-tailed Student’s *t* test (**C**), and 2-way ANOVA with Bonferroni’s (**D**–**F**). *P < 0.05; **P < 0.01; ***P < 0.001.

Although effective in multiple cell and preclinical mouse models, we acknowledge that 3-MA may not be ideally suited for human use because of its broad impact on cell cycle–related pathways. We therefore aimed to identify individual 3-MA targets that could elicit a similar tumor-selective phenotype and could, thus, be exploited as a therapeutic target. Since 3-MA is reported to inhibit the Class III PI3-kinase VPS34, we first asked if VPS34 inhibition could phenocopy 3-MA treatment. Dose-response experiments were performed using VPS-IN1, a VPS34 inhibitor (35), on T4-KPC and MS1 cells. While we observed a decrease in T4-KPC cellular proliferation at 10 μM VPS-IN1, we also observed cytotoxicity in normal pancreas cells at the same — and lower — doses (Supplemental Figure 5, A and B). To explore potentially novel mechanisms of 3-MA function, we identified and ranked DEGs from highest to lowest significance and identified *Cdhr2*, *Ptk2*, *Nedd9*, *Mcu*, and *Perp* as the top significantly altered DEGs upon 3-MA treatment (Figure 5A). Out of these 5 genes, *Perp* was the only gene associated with a survival disadvantage among Pancreatic ductal adenocarcinoma (PDAC) cancer patients archived in The Cancer Genome Atlas (TCGA) (*P* = 0.0000527) (Figure 5B). We subsequently determined that (a) *Perp* expression was significantly decreased in tumors from 3-MA–treated KPC mice compared with vehicle-treated controls (Figure 5C), (b) *Perp* was reduced in the tumor and the muscles of PDO mice treated with 3-MA (Supplemental Figure 6A), and (c) *Perp* was increased in skeletal muscle samples from young and aged tumor-bearing mice (Supplemental Figure 6B).

Our data suggest, in the context of pancreatic cancer, that *Perp* may promote tumor growth. To test this hypothesis more rigorously, we next aimed to determine the impact of *Perp* reduction in cancer cell lines. Baseline comparison of *Perp* gene expression across cultured cell lines revealed higher overall levels of *Perp* in PDAC cell lines (T4-KPC and T3-KPC) compared with MS1. 3-MA treatment significantly reduced *Perp* expression in T4-KPC and T3-KPC cell lines (Figure 5D). We also observed an increase in Perp expression in other nonpancreas cancer cell lines — lung cancer cell line (393P) and breast cancer cell line MCF-7) — in comparison to a normal human pancreatic epithelial cell line (HPNE). 3-MA successfully decreased Perp expression in 393P cells but not in MCF-7 cells (Supplemental Figure 6C).

Consistent with cytotoxicity data, Vps34 inhibition did not reduce increased *Perp* expression, indicating that 3-MA–mediated Perp reduction is Vps34 independent (Supplemental Figure 6D). We next stably reduced *Perp* expression in PDAC cell lines (T4-KPC and T3-KPC) using lentiviral shRNAs (Figure 5E). Consistent with 3-MA effects, *Perp* knockdown decreased cellular proliferation in T4-KPC and T3-KPC cells (Figure 5F). Together, these data support the hypothesis that, as opposed to VPS34 inhibition, 3-MA elicits antitumor effects by reducing *Perp* expression. Finally, we observed upregulated p53 and senescence-associated pathway activity in *Perp*-knockdown tumor cells, which may provide insight into Perp-dependent mechanisms of tumor growth inhibition (Supplemental Figure 6, E and F).

### Perp is increased in pancreatic cancer patient samples.

**Figure 6:**
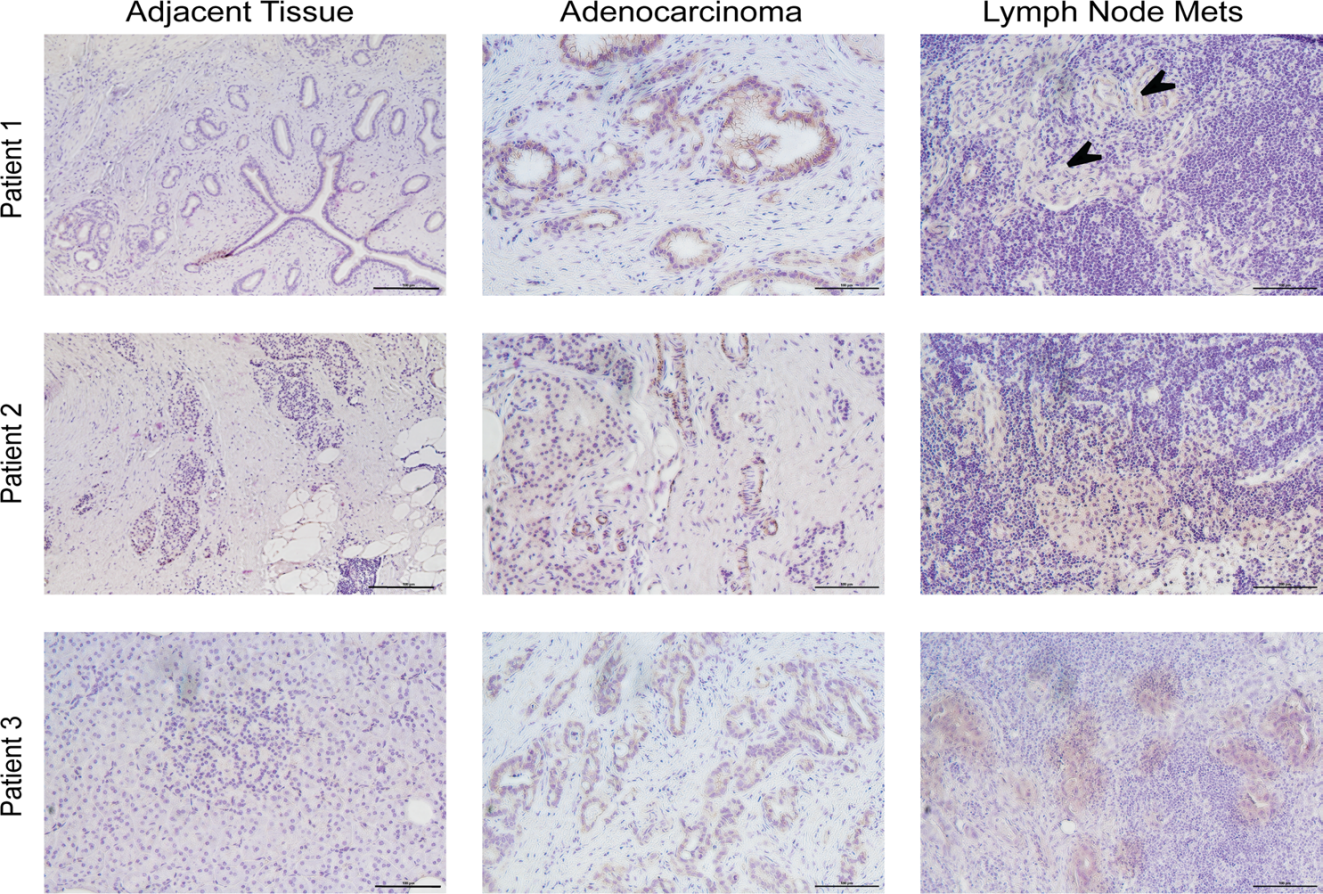
PERP is elevated in human pancreatic cancer. Representative images of PERP staining in tumor-adjacent tissue, adenocarcinoma, and lymph node metastatic nodules of the same patient (3 representative patients shown, *n* = 10 patients analyzed). Arrowheads, brown staining for PERP protein detected in lymph node mets. Scale bar: 100 μm.

Considering the robust expression of *Perp* in cancer cell lines, our observations that 3-MA reduced *Perp* expression in vitro and in vivo, and that shRNA-mediated *Perp* reduction attenuated tumor cell proliferation, we next wanted to determine the extent to which *Perp* expression was associated with human pancreatic cancer. Tissue samples were collected from 10 patients at resection and encompassed PDAC, tumor adjacent, and metastatic regions. Anti-PERP immunostaining revealed light to minimal staining in tumor-adjacent tissue, intense staining of primary PDAC lesions, and diffuse PERP reactivity in metastatic tissues (Figure 6). Adjacent tissues containing histologically abnormal lesions also stained positive for PERP (Supplemental Figure 6G).

Next, we utilized tumor tissue microarrays (TMAs) to query PERP expression in tumor samples from 200 unique pancreas adenocarcinoma patients (Supplemental Figure 7B and Supplemental Table 1). These patients were all eligible for tumor resection and were administered gemcitabine as an adjuvant therapy. PERP expression, as determined by IHC staining of TMAs, was summarized using the H-Score (36, 37) which is a function of the strength of staining (0, negative; 1, weak; 2, moderate; 3, strong) multiplied by the percent of cells staining (0%–100%) for that intensity, yielding a continuous score that can range in this instance from 0 to 300 for each sample stained. The multiple core-level H-Scores were averaged to generate a single, per-subject PERP measure for use in subsequent analyses. As seen in Supplemental Table 2, individuals with “low” expression (Average PERP ≤ 25.0) were comparable with those with “high” expression (PERP > 25) for all patient demographic variables considered. Individuals with low expression exhibited a modestly reduced, but significant, rate of patient-reported pancreatitis (22.0% versus 38.9%, *P* = 0.0187) when compared with patients with higher PERP expression (Supplemental Table 2). While survival differences between high and low PERP cohorts did not reach statistical significance (663 versus 588 days, *P* = 0.4050; hazard ratio [HR] = 1.13, 95% CI, 0.85–1.52), studies with larger patient cohorts are warranted, given observed data trends after adjusting for age, sex, obesity, and patient-reported DM (Supplemental Figure 7, A and B, and Supplemental Table 3).

### Perp depletion negates 3-MA–mediated antitumor and prosurvival benefits.

**Figure 7:**
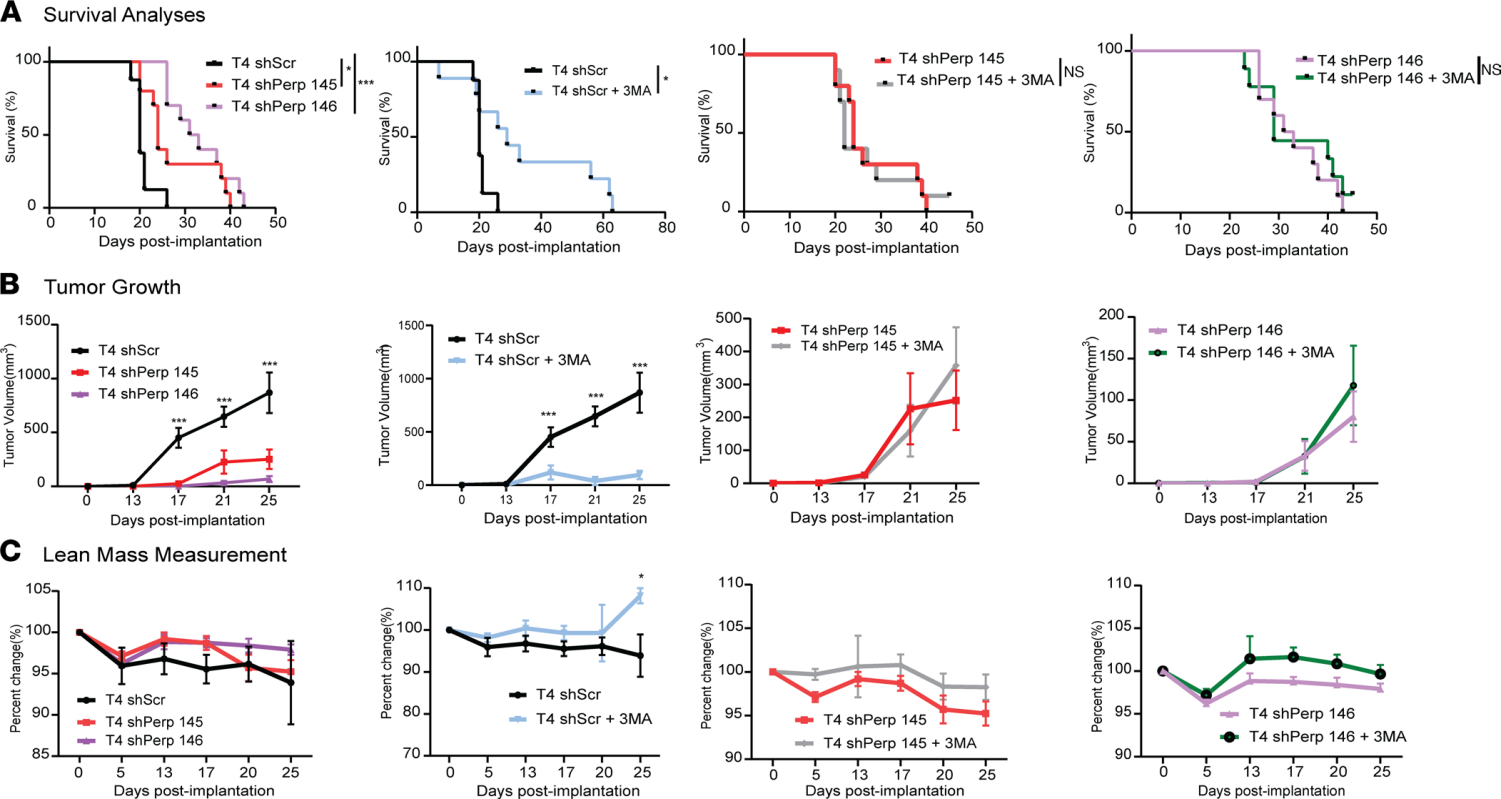
Perp inhibition decreases tumor growth but does not rescue muscle wasting in vivo. (**A**–**C**) Survival analyses, tumor volume, lean mass measurements of T4 shScr, T4 shPerp 145, T4 shPerp 146, T4 shScr ± 3-MA, T4 shPerp 145 ± 3-MA, and T4 shPerp 146 ± 3-MA (*n* = 8 for T4shScr and *n* = 10 for T4shPerp 145/146 ± 3-MA). Data are shown as mean ± SEM and compared using Log-rank test (**A**), or 2-way ANOVA with Bonferroni correction (**B** and **C**). *P < 0.05; ***P < 0.001.

We next sought to determine the extent to which Perp mediates the antitumor and prosurvival effects of 3-MA treatment. T4 shScr, T4 shPerp 145, and T4 shPerp 146 cells were transplanted into aged (78 weeks) mice and treated with vehicle or 3-MA. Compared with T4 shScr control mice, T4 shPerp 145 and T4 shPerp 146 tumor–bearing mice exhibited a significant survival advantage (Figure 7A). While 3-MA extended survival in control T4 shScr–bearing mice (Figure 7A), there was no survival advantage observed in T4 shPerp 145 and T4 shPerp 146 tumor–bearing mice treated with 3-MA (Figure 7A). Consistent with a decrease in tumor cell proliferation upon Perp knockdown (Figure 5F), we observed a marked decrease in tumor volume and progression in mice bearing T4 shPerp 145 and T4 shPerp 146 tumor cells as compared with T4 shScr mice (Figure 7B). Whereas 3-MA was able to significantly slow in vivo tumor progression in control mice, T4 shPerp 145 and T4 shPerp tumor progression was not affected by 3-MA administration (Figure 7B). In the absence of 3-MA treatment, we did not observe longitudinal differences in lean mass between groups (T4 shScr, T4 shPerp 145, T4 shPerp 146) (Figure 7C). We did, however, find that 3-MA significantly rescued lean mass of mice bearing T4 shScr tumors and did not rescue lean mass in T4 shPerp 145 or T4 shPerp 146 tumor–bearing mice (Figure 7C). Fat mass was not significantly altered in mice bearing T4 shPerp 145 and T4 shPerp 146 tumors, and 3-MA treatment did not further alter these trends (Supplemental Figure 8A). There were no significant differences in overall body weight, although due to tumor burden, weight is a significant confounding variable (Supplemental Figure 8B). On a molecular level, we observed significant decreases in *Trim63*, *Fbxo32*, and *Perp* in muscles of mice bearing T4 shPerp 145 and T4 shPerp 146 tumors compared with control T4 shScr tumor mice. This significant downward trend was furthered in the presence of 3-MA (Supplemental Figure 8C). This suggests that 3-MA may play a role in preservation of muscle mass independently of its effect on tumor progression. Taken together, these data show that *Perp* is a positive regulator of in vivo tumor progression and provide strong evidence that *Perp* is a significant 3-MA target.

## Discussion

Pancreatic cancer is one of the most fatal late-onset cancer subtypes. Late detection, poor therapeutic efficacy, and rapid physiological decline are all contributing factors. Cancer cachexia, which encompasses muscle weakness, fatigue, anorexia, respiratory, and cardiac failure, is particularly problematic in pancreatic cancer (38). Although many groups have made efforts to identify therapeutic interventions to control or reverse cancer cachexia, current clinical management mostly entails nutritional and hormonal supplementation, to minimal success. A major roadblock hampering clinical cachexia management appears to be that, while there are many targets and compounds demonstrating efficacy in preclinical models, many fail to meet primary endpoints in clinical trials (6). We posited that one contributing factor might be the use of young mice for the majority of wasting/cachexia studies. Six- to 8-week-old mice translate to ~20 years in human age, while the median age of diagnosis for pancreatic cancer is ~65–70 years. To address this issue, we utilized mice of 78–80 weeks (corresponding to 60+ years in humans) to study cancer cachexia, an age more aligned with patients. To our surprise, we noticed substantial molecular differences in control and wasting muscles by simply changing one experimental variable (age). We acknowledge that age is just one variable that can be considered in order to develop models that better capture the human condition. Other clinical variables that would be interesting to incorporate into existing model systems include stress, chemotherapy, and other palliative/supportive interventions.

A key element that likely impacts the translational relevance of preclinical cachexia models is the generation and analysis of site-specific tumors. Though many studies still utilize s.c. tumor cell injection paradigms, orthotopic approaches generally permit more accurate recapitulation of key variables such as the tumor microenvironment. While autochthonous tumor models such as KPC offer the same advantages (and more) due to slower tumor development, these models require considerably more resources and are often difficult to manage from an intervention standpoint, given variabilities in tumor onset and the heterogeneity in the timing/severity of cachexia. In this regard, orthotopic transplantation–based approaches are ideally suited to model and study cancer cachexia. As to the cell types used for these orthotopic studies, there are several further issues to consider. The most common type of cells used are mouse tumor cells implanted into syngeneic, immunocompetent mice — an approach that best permits analysis of tumor/cachexia development in a normal physiological environment. In this study, we primarily utilized orthotopic implantations of KPC pancreatic tumor cells into syngeneic C57BL/6J mice to generate site-appropriate tumors in mice with an intact immune system. Since we also wanted to determine if tumor cells of human origin would also respond similarly to 3-MA, we weighed 2 options: (a) implantation of immortalized human cancer cell lines into athymic nude mice or (b) implantation of PODs into NOD mice. While the former option is straightforward and reproducible, the latter option offers the advantage of studying actual patient tumors, maintained in a 3-D state while in culture, in vivo. Our data show that this approach is a viable model for generating pancreatic tumors. We were then able to use this model to corroborate our fully mouse-based (KPC) observations using human cells. In all, we utilized diverse model systems including in vitro cell models, aged and young KPC-based murine models, POD/NOD mouse models, and patient tumor tissues to query cachexia, tumor progression, and 3-MA/*Perp* mechanisms of action. By taking a diversified approach, we contend that *Perp* has strong translational potential as a pancreatic cancer biomarker and/or therapeutic target.

We identified 3-MA via transcriptomics and pathway analyses of skeletal muscle from control and tumor-bearing mice. This identification was based on altered expression of PI3K and autophagy-associated transcripts in wasting aged muscle. While we had expected to similarly observe autophagy-related pathway alterations in tumor cells, gene expression profiling of KPC cells with or without 3-MA suggested otherwise. In the absence of a clear autophagy signature, we took a candidate-based approach to probe mechanisms underlying 3-MA–associated tumor cell cytotoxicity. We made the following key observations. First, *Perp* reduction was able to phenocopy key 3-MA outcomes, including selectivity for tumor cells compared with normal cells and stronger effects in tumor cells of pancreatic versus lung origin. Second, *Perp* expression was consistently higher in tumor cells/samples compared with controls, and 3-MA was able to reduce *Perp* expression in all contexts tested. Third, Perp knockdown in PDAC cell lines led to a decrease in tumor progression, implying that Perp has tumor-promoting characteristics. Fourth, 3-MA was unable to further reduce proliferation of Perp-knockdown cell lines, thus establishing Perp as a critical 3-MA target. Fifth, we observed that decreases in Perp-knockdown tumor progression did not entirely correlate with lean mass preservation, though it did lead to a decrease in atrophy gene expression. One explanation might be the activation of alternative pathways such as autophagy. Another explanation might be that, while inhibition of Perp does decrease tumor cell proliferation/in vivo tumor progression, it might be altering the tumor microenvironment or the tumor secretome, which in turn sustains the cachectic phenotype. While unexpected, this observation is consistent with other reports suggesting that lean mass/muscle loss is not solely dependent on tumor size (39, 40) and imply that there are size- and Perp-independent mechanisms underlying muscle loss in these KPC tumors. Finally, we noted that 3-MA treatment led to further suppression of atrophy genes in the muscles of T4-KPC shPerp 145 and T4-KPC shPerp 146 tumor–bearing mice — with no additional decrease in tumor size. This result supports the hypothesis that 3-MA has a direct muscle preservation effect independent of its effect on tumor progression. These observations — coupled with our findings that VPS34 inhibition was equally, if not more, cytotoxic to control (MS1) versus tumor cells and was unable to inhibit *Perp* expression — point to a mechanism of 3-MA action. Still, further work is needed to elucidate the precise relationship between 3-MA and *Perp* and to determine the extent to which *Perp* contributes to tumor progression.

A challenge in the cachexia field is identifying antiwasting interventions that do not promote tumor progression or, ideally, simultaneously inhibit tumor growth. There are many contexts in which targeting cachexia may aggravate the tumor; one such example is a recent report showing that an increase in muscle oxidative stress rescues muscle atrophy (22). Targeting that same pathway in tumor cells, however, can promote tumor aggressiveness and metastasis (41), thus diminishing the potential of this approach as a systemic intervention. That said, there is a limited number of studies demonstrating that the same therapy can inhibit cachexia and antagonize cancer progression (23). Here, we show that 3-MA can inhibit tumor progression. Moreover, we show that 3-MA inhibits *Perp* in both contexts and that *Perp* reduction is sufficient to antagonize pancreatic cancer cell growth. These data suggest that *Perp* is a key molecular mediator of 3-MA in the tumor and further imply that *Perp* may have tissue- or context-specific functions. More work is needed to better understand this effect.

Our discovery of a compound that dually inhibits tumor progression and limits muscle wasting was serendipitous. This would not have been possible without the comparative analysis of young versus aged KPC cancer cachexia models. Thus, our study underscores the importance of model selection and makes a case that concerted efforts need to be made toward developing and studying cachexia models that more faithfully recapitulate the human condition. A major finding from these studies was the identification of *Perp* as a potentially novel 3-MA target and putative oncogene in the context of PDAC. PERP protein expression in a limited human PDAC cohort highlighted the prognostic potential of *Perp*, a potential that should be explored in larger patient data sets. Unfortunately, there are currently no specific inhibitors for PERP. Identification and/or development of such compounds would be an exciting next step toward advancing therapies that target both muscle wasting and tumor progression; these therapies compose the ideal scenario for treating aggressive, cachexia-promoting tumors like pancreatic cancer.

## Methods

### Animal studies.

C57BL/6J and NOD.Cg-Prkdcscid/J (referred to as NOD mice) mice were obtained from The Jackson Laboratory. Young (8 weeks) and aged (78 weeks) male C57BL/6J mice (The Jackson Laboratory) were used for orthotopic implantations. In total, 0.5 × 10^4^ T4-KPC cells were injected into the mouse pancreas. After necropsy, tumor tissue and muscles were flash frozen in liquid nitrogen or formalin fixed for further analysis.

For 3-MA studies, 0.5 × 10^4^ T4-KPC cells were injected into the mouse pancreas. After 7 days of implantation, mice were randomized into 2 groups: vehicle or 3-MA–treated. 3-MA (30 mg/kg) was dissolved in saline and injected via i.p. to tumor-bearing mice once weekly. Saline was used as a vehicle control. Knockdown (RNA interference) studies: 0.5 × 10^4^ T4 shScr, T4 shPerp 14, and T4shPerp 146 were injected in 78-week-old C57BL/6J male mice. 3-MA treatment protocols were performed as described above.

Human pancreatic cancer organoids were a gift from Martin Fernandez-Zapico (Mayo Clinic). Approximately 100 organoids were injected in the pancreas of NOD mice. After 10 days of implantation, mice were randomized into 2 groups; the experimental group was i.p. injected weekly with 3-MA as described above, while the control group received saline (vehicle) injections.

### EchoMRI imaging.

EchoMRI Body Composition Analyzer (Echo Medical Systems) was used for longitudinal body composition analyses as previously described (42). Mice were regularly measured for lean mass, fat mass, and body weight.

### Cell culture and reagents.

Pancreatic cancer cell lines (T4- and T3-KPC cells) were derived from KPC mice as previously described (43) and were a gift from David Tuveson (Cold Spring Harbor Laboratory, Long Island, New York, USA). MS1, a mouse endothelial pancreas cell line, and CFPAC were obtained from ATCC. The Kras^LA1/+^p53^R172H/Δg/+^ lung adenocarcinoma cell line (393P) was generated as previously described (44). All cell lines were cultured in DMEM (Thermo Fisher Scientific) with 10% FBS, 100 IU/mL penicillin, and 100 μg/mL streptomycin, and incubated at 37°C in a humidified incubator with 5% CO_2_. C2C12 myoblasts were purchased from ATCC and cultured in DMEM with 10% FBS until confluent. After reaching confluency, the myoblasts were differentiated in DMEM with 2% horse serum and 1 μg/mL insulin for 72 hours, as previously described (22). 3-MA (item no. 13242) for in vitro and in vivo studies was purchased from Cayman Chemical.

### Cancer cell CM preparation.

KPC cell lines were seeded and cultured in DMEM with 10% FBS, as previously described (22). Upon reaching 70% confluency, cells were washed twice with 1× PBS and cultured in serum-free DMEM for 24 hours. The media was then collected and centrifuged at 1200*g* for 10 minutes at 37°C, and the supernatant was collected in a fresh tube to be either used immediately or stored at −80°C for future use. CM was prepared from equal number of cancer cells for each cell line. CM was reconstituted with 2% horse serum 1 μg/mL insulin before treating myotubes.

### Lentiviral transduction.

Lentiviral transduction (transfection, virus collection, target cell infection) was carried out as previously described (22). Short hairpin RNA (shRNA) constructs for stable knockdown of *Perp* were obtained from Sigma-Aldrich (TRCN0000112146, TRCN0000112145). A scrambled shRNA construct was obtained from Addgene (catalog 1864) and used as a negative control. T4-KPC and T3-KPC cells were incubated with lentivirus for 24 hours, followed by puromycin selection.

### Cell viability assays.

Cell proliferation and cell death were measured by live cell analysis (Incucyte ZOOM Live-Cell Imaging System, Essen Bioscience) as previously described (42).

### RNA isolation and qPCR.

Total RNA was extracted from cells or tissue lysates by using TRIzol reagent (Invitrogen) as previously described (22) and was isolated using RNeasy columns (Qiagen), as per the manufacturer’s protocol. cDNA was synthesized using cDNA synthesis kit (Applied Biosystems) according to the manufacturer’s protocol. qPCR was performed using SYBR Green master mix (Bio-Rad). *Tubulin* was used as an internal control. Relative gene expression analysis was performed by using the ΔΔCt method, as described previously (22).

### RNA-Seq analyses.

RNA extracted from cells and tissues was submitted to the Mayo Clinic Medical Genome Facility, where RNA quality was determined using the Fragment Analyzer from AATI. Library preparation, sequencing, and analyses were performed as described previously (42). The accession nos. are PRJNA773714, PRJNA773111, PRJNA773410 on NCBI SRA (https://www.ncbi.nlm.nih.gov/sra).

### Immunostaining.

Murine muscle tissue for immunostaining was placed in a sucrose sink (30%) overnight prior to freezing and sectioning. Sections (8–10 μm) were postfixed in 4% paraformaldehyde (PFA) for 5 minutes at room temperature prior to immunostaining. Once fixed, tissues were stained with rat anti-laminin (MilliporeSigma, 4HB-2) as previously described (42). C2C12 myotubes were treated with CM for 24 hours and stained with Myosin heavy chain antibody (MF20, Developmental Studies Hybridoma Bank). Secondary antibodies were Alexa Fluor conjugates (488 or 647) from Invitrogen (catalogs A21202 and A21247).

### IHC.

IHC was performed as described previously (22). Pancreatic tumor sections prepared from human patient tissues were stained with PERP antibody at a dilution of 1:25 (Novus Biologics, catalog NBP2-75616) using the VECTASTAIN Elite ABC-HRP Kit (Vector Laboratories) per manufacturer’s instructions. The sections were scored for intensity and extent by Lizhi Zhang (Mayo Clinic, Rochester, Minnesota, USA). H&E staining of tumor-organoid sections was performed by the Mayo Clinic Histology Core Facility (Phoenix, Arizona, USA).

### Statistics.

Data are represented as the mean ± SEM using GraphPad Prism (GraphPad Software) unless noted otherwise in the figure legends. Quantification of muscle CSA and minimum feret diameter were analyzed by nonlinear regression (least-squares method) and compared between conditions using an extra-sum-of-squares F test. All in vitro experiments were repeated at least 3 times or as indicated in the figure legends. Graphical abstract and illustrative schematics were made utilizing BioRender.

### Study approval.

Resected tumor specimens from pancreatic cancer patients were obtained under a study titled “Development of a Pancreatic Cell Line Bank to Support Pancreatic Cancer Research.” The tissues were collected with appropriate consent under the Mayo Clinic IRB (no. 66-06). The fresh tumor specimens were collected and brought to the laboratory for processing, culturing, and propagation of pancreatic cancer organoid cell lines. The organoids that were established in the lab were assigned a lab number different from the subject number. The subject number was not shared outside the laboratory. Histological slides of paraffin-embedded human tissue from 10 distinct, deidentified patients include matched tumor-adjacent, PDAC, and metastasis tissue and was obtained from the Mayo Clinic SPORE in Pancreatic Cancer. All patients provided written informed consent, and the study was approved by the Mayo Clinic IRB. All animal experiments performed in this study were approved by the Mayo Clinic IACUC.

All animal experiments performed in this study were approved by the Mayo Clinic IACUC.

## Author contributions

AD and JDD planned the experimental design. AD performed experiments and analyzed data. PCAW, RES, DSC, and AMD provided technical assistance. TLH and EWK assisted with patient and organoid samples. WRB performed patient statistical analyses. LZ performed patient tumor scoring. GLR and MEFZ contributed reagents and patient samples. AD and JDD wrote the manuscript.

## Supplementary Material

Supplemental data

## Version 1 12/07/2021

In-Press Preview

## Version 2 01/25/2022

Electronic publication
