# Supplementary material for: Anticachectic regulator analysis reveals Perp-dependent antitumorigenic properties of 3-methyladenine in pancreatic cancer
Source: JCI Insight. 2022 Jan 25;7(2):e153842. doi: 10.1172/jci.insight.153842 (PMC8855816; doi:10.1172/jci.insight.153842)
Supplement: Supplemental data [file jciinsight-7-153842-s122.pdf]

Supplementary Material

Anti-cachectic regulator analysis reveals novel Perp-dependent anti-tumorigenic properties of 3-methyladenine in pancreatic cancer

Aneesha Dasgupta[1], Paige C. Arneson-Wissink[1], Rebecca E. Schmitt[1], Dong Seong Cho[1], Alexandra M. Ducharme[1], Tara L. Hogenson[2], Eugene W. Krueger[1,3], William R. Bamlet[4], Lizhi Zhang[5], Gina Razidlo[1,3], Martin E. Fernandez-Zapico[2], Jason D. Doles[1]*

[1]Department of Biochemistry and Molecular Biology

[2]Schulze Center for Novel Therapeutics, Division of Oncology Research, Department of Oncology

[3]Division of Gastroenterology and Hepatology

[4]Health Sciences Research

[5]Department of Laboratory Medicine and Pathology

Mayo Clinic, Rochester, Minnesota, 55905 USA

*Corresponding Author:

Jason D Doles
Department of Biochemistry and Molecular Biology
Mayo Clinic
200 First St SW
Guggenheim 16-11A1
Rochester, MN 55905
Tel: (507) 284-9372
Fax: (507) 284-3383
E-mail: Doles.Jason@mayo.edu

Supplementary Table 1: Patient Demographics

	(N=200)	
Age at Diagnosis	63.1	(11.4)
Sex: Male	113	(57%)
Body Mass Index (BMI)	28.5	(5.5)
Obesity (BMI ≥ 30)	62	(31%)
Patient Reported Diabetes	65	(33%)
Patient Reported Pancreatitis,	48	(29%)
Grade		
2	17	(9%)
3	133	(72%)
4	36	(19%)
Positive nodes	130	(65%)
Vital Status: Deceased	184	(92%)
PERP Average Histoscore *	25.0	(0, 300.0)
PERP – Median Dichotomized		
Low (≤ 25.0)	108	(54%)
High (> 25.0)	92	(46%)

Categorical variables are summarized with Frequency (%).

Continuous variables are summarized with mean (sd) or median (min, max)*

Supplementary Table 2: Perp Average Intensity Median Dichotomized

	Low (≤ 25.0) (N=108)	High (> 25.0) (N=92)	p value
**Age at Diagnosis**			0.8647
Mean (SD)	63.2 (11.8)	63.0 (10.9)	
**Sex**			0.7704
Female	48 (44.4%)	39 (42.4%)	
Male	60 (55.6%)	53 (57.6%)	
**Obesity (BMI ≥ 30)**			0.1981
Missing	0	2	
No	70 (64.8%)	66 (73.3%)	
Yes	38 (35.2%)	24 (26.7%)	
**Patient Reported Diabetes**			0.9758
No	73 (67.6%)	62 (67.4%)	
Yes	35 (32.4%)	30 (32.6%)	
**Patient Reported Pancreatitis**			0.0187
Missing	17	20	
No	71 (78.0%)	44 (61.1%)	
Yes	20 (22.0%)	28 (38.9%)	
**Grade**			0.2404
Missing	10	4	
2	11 (11.2%)	6 (6.8%)	
3	72 (73.5%)	61 (69.3%)	
4	15 (15.3%)	21 (23.9%)	
**Positive nodes**			0.1219
No	43 (39.8%)	27 (29.3%)	
Yes	65 (60.2%)	65 (70.7%)	

Supplementary Table 3: PERP (Median dichotomized) – Survival

	Event/Total	Median (95% CI)[1]	Unadjusted HR (95% CI)[2]	P-value	Adjusted HR (95% CI)	p-value
**PERP (Median)**				0.4050[3]		0.2521[4]
0: Low (≤ 25.0)	99/108	663.0 (604.0-879.0)	--		--	
1: High (>25.0)	85/92	588.0 (498.0-849.0)	1.13 (0.85-1.52)		1.19 (0.88-1.61)	

Supplementary Figure 1

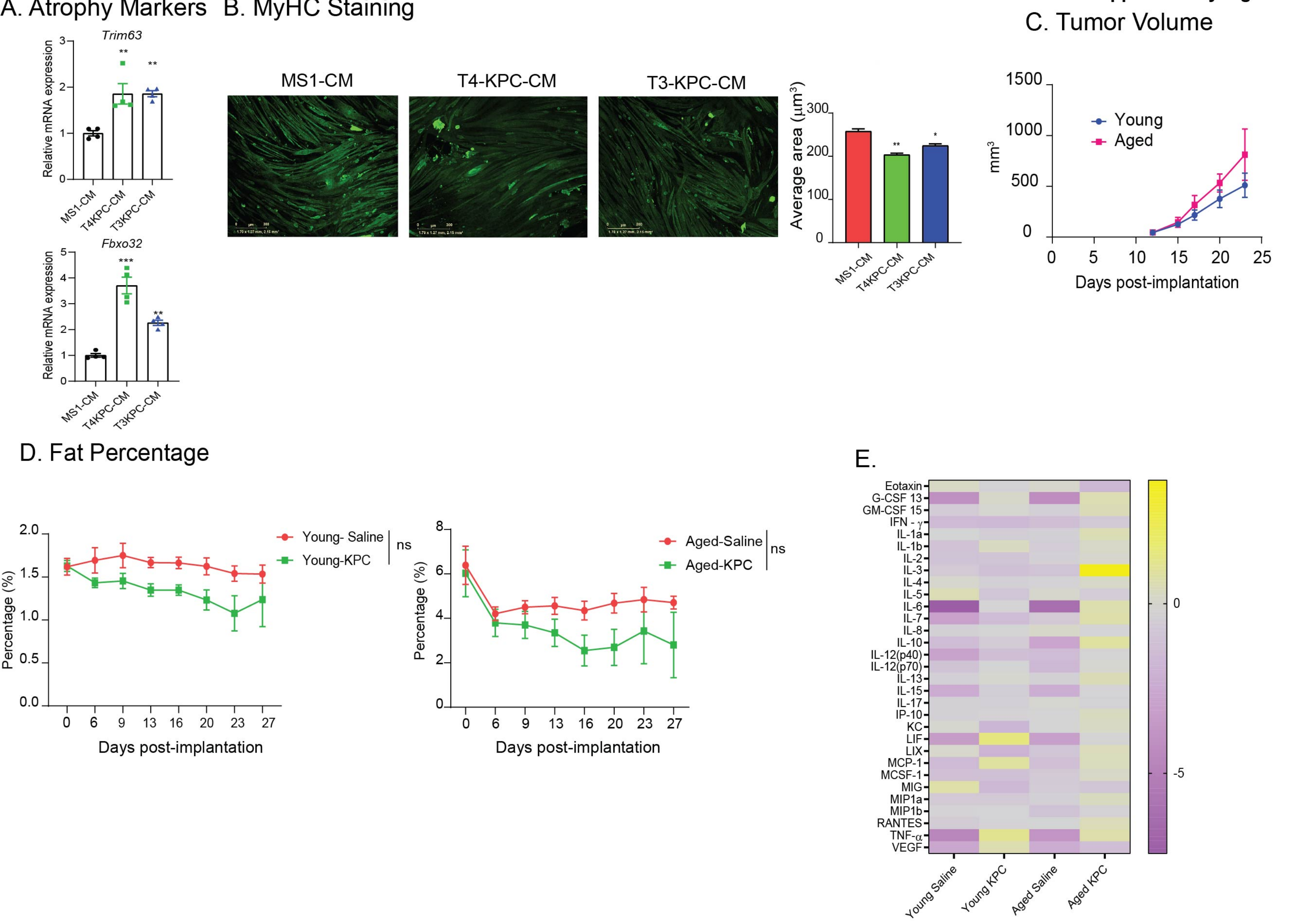

Supplementary Figure 1: KPC pancreatic cancer cells promote cachectic phenotypes *in vitro* and *in vivo*. A-B. mRNA expression of *Trim63* and *Fbxo32*, MyHC immunostaining and quantification in C2C12 myotubes treated with T4-KPC and T3-KPC CM. C. Longitudinal analyses of tumor volume in the young and aged tumor-bearing mice. D. Longitudinal analyses of fat mass in control and tumor-bearing mice. E. Post-necropsy serum cytokine levels in young and aged cohorts (n=5 in each group). Data is mean ± SEM compared with one-way ANOVA with Bonferroni's test (A-B), two-way ANOVA with Bonferroni's test (D). *p<0.05; **p<0.01; ***p<0.001.

Supplementary Figure 2

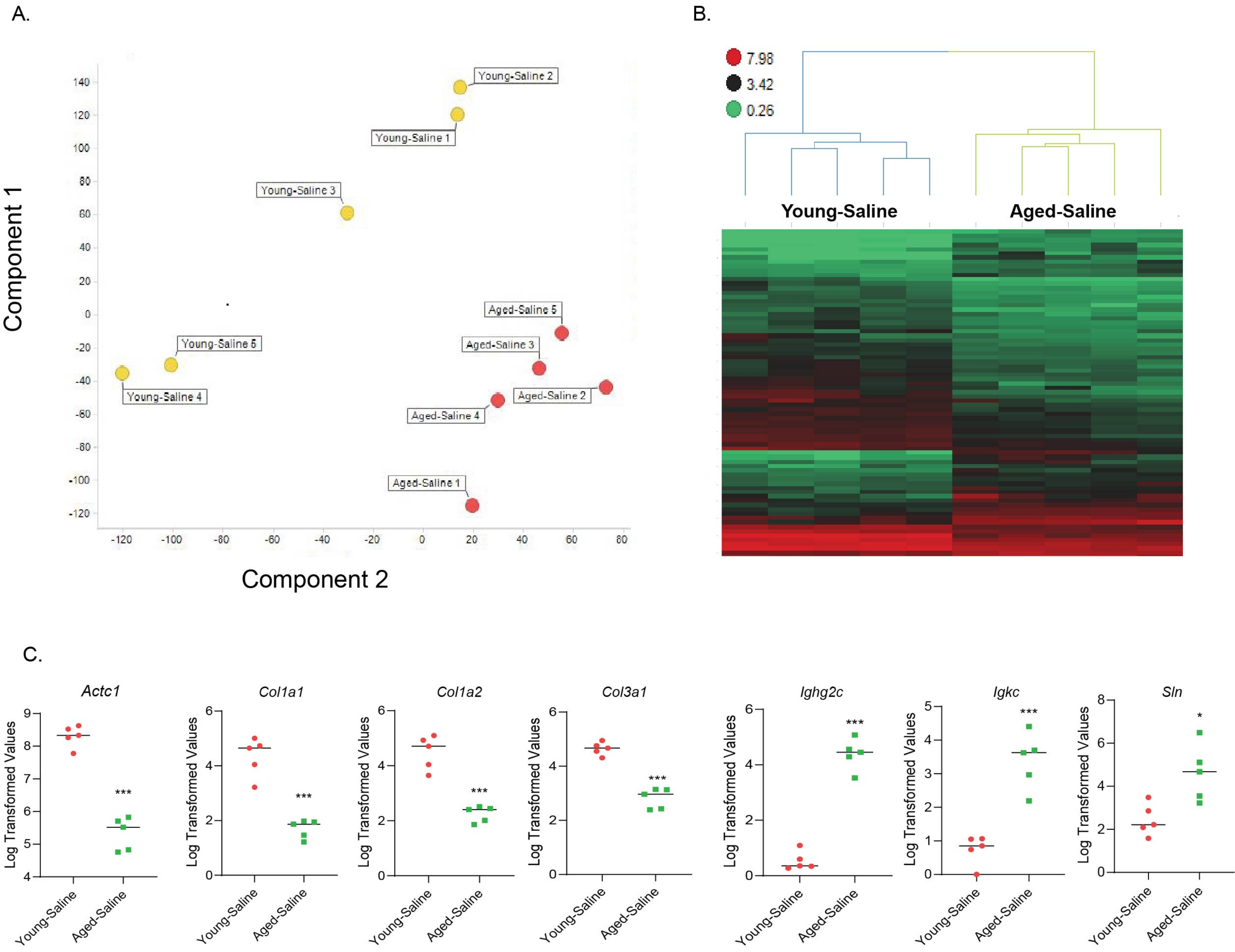

Supplementary Figure 2: Comparative gene expression analysis of young/aged muscle and 3-MA treated tumors. A. A principal component analysis (PCA) plot depicting young and aged control muscle transcriptomes. B. A heatmap of DEGs and hierarchical clustering analysis of young versus aged control muscle. C. Log transformed and row normalized FPKM values of selected DEGs – Actin alpha cardiac muscle 1 (*Actc1*), Collagen type 1 alpha 1 and 2 (*Col1a1* and *Col1a2*), Collagen type 3 alpha 1 (*Col3a1*), immunoglobulin heavy constant gamma 2c (*Ighg2c*), Immunoglobulin kappa constant (*Igkc*), Sarcolipin (*Sln*). DData is mean ± SEM compared with Student's t-test (C).

Supplementary Figure 3

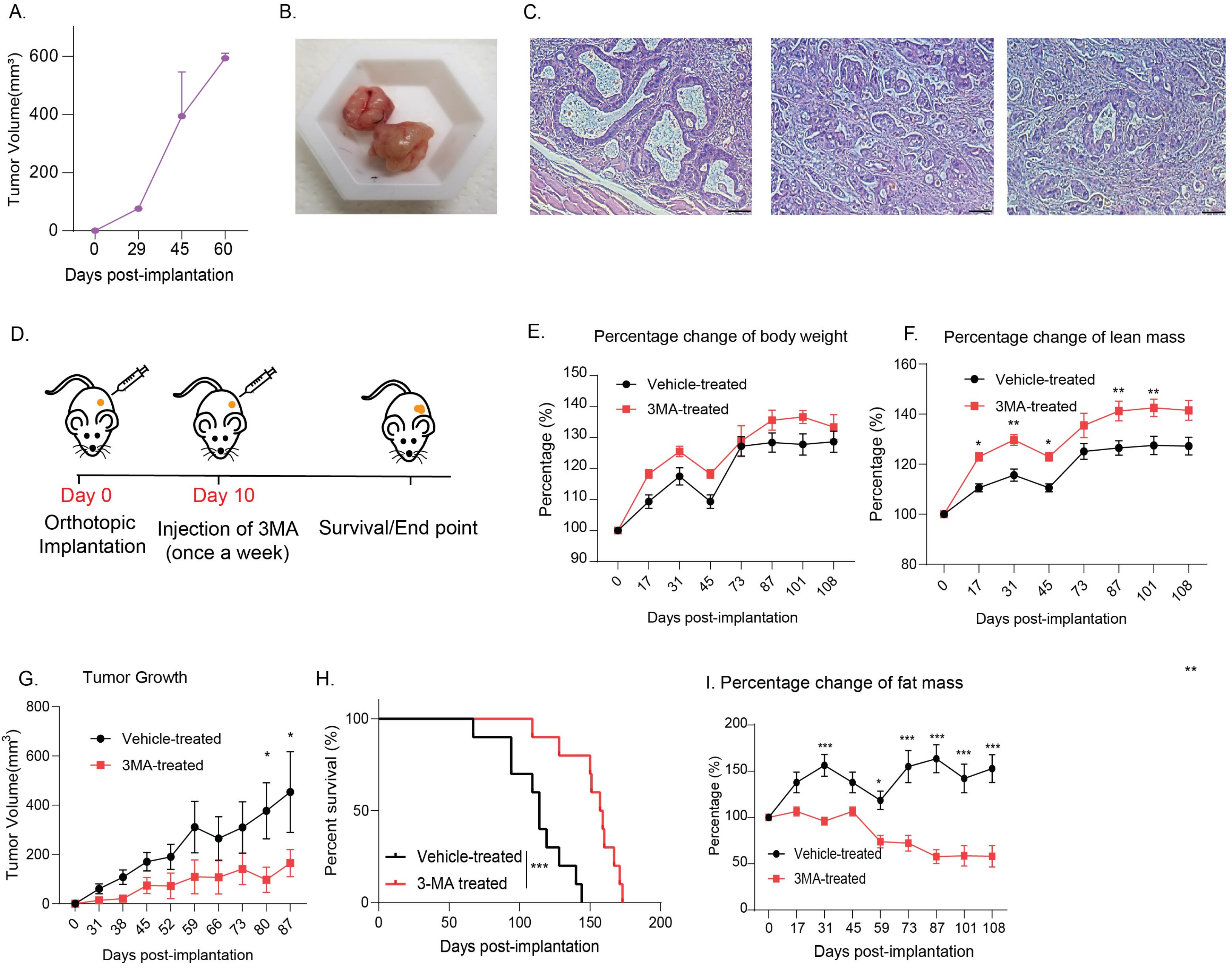

Supplementary Figure 3: *In vivo* analyses of PDO-implanted mice treated with 3-MA. A. Quantification of tumor volume post-PDO implantation (n=3). B. Post-necropsy picture of tumor-organoids. C. Representative images of hematoxylin and eosin staining of tumor-organoid sections (n=3). Scale bar is 100μm. D. Schematic illustration of vehicle (n=10) and 3-MA (n=10) treatments in tumor- organoid bearing mice. E-G. Longitudinal analyses of body weight, lean mass and tumor growth in PDO mice +/- 3-MA. H. Survival analyses of vehicle and 3-MA treated PDO mice. I. Longitudinal analyses of fat mass in tumor-organoid bearing mice treated with 3-MA. Data is mean ± SEM, compared with 2-way ANOVA with Bonferroni's (E-G), Log rank Mantel-Cox test (H). *p<0.05; **p<0.01; ***p<0.001.

Supplementary Figure 4

Altered genes in *in vivo* tumor validated by qPCR

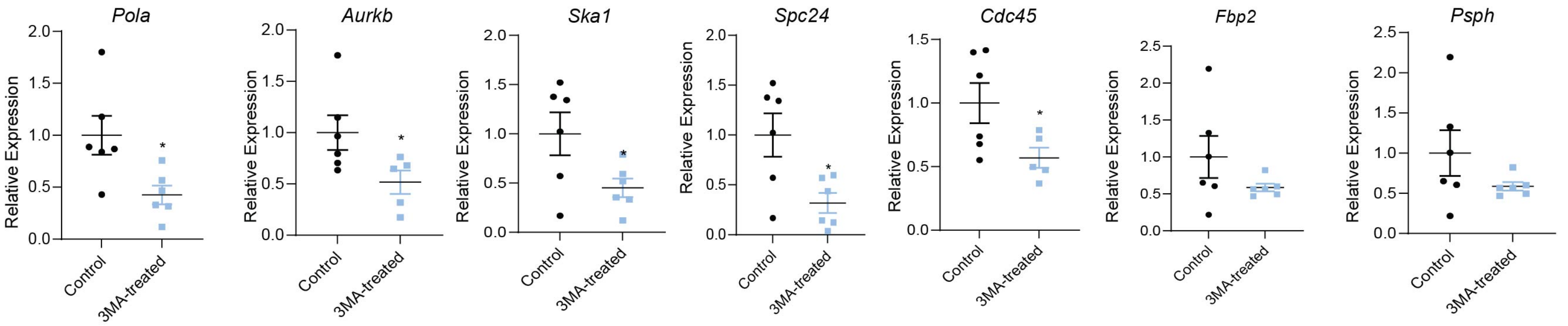

Supplementary Figure 4: Validation of genes altered *in vivo*. mRNA expression of DEGs in the tumors of mice treated with and without 3MA – DNA polymerase (*Pola*), Aurora kinase B (*Aurkb*), Spindle and kinetochore associated protein 1 (*Ska1*), Kinetochore protein Spc24 (*Spc24*), Cell division cycle 45 (*Cdc45*), Phosphoserine phosphatase (*Psph*), Fructose-1,6-bisphosphate (*Fbp2*). Data is mean ± SEM compared with Student's t-test. $^*p<0.05$; $^{**}p<0.01$; $^{***}p<0.001$.

Supplementary Figure 5

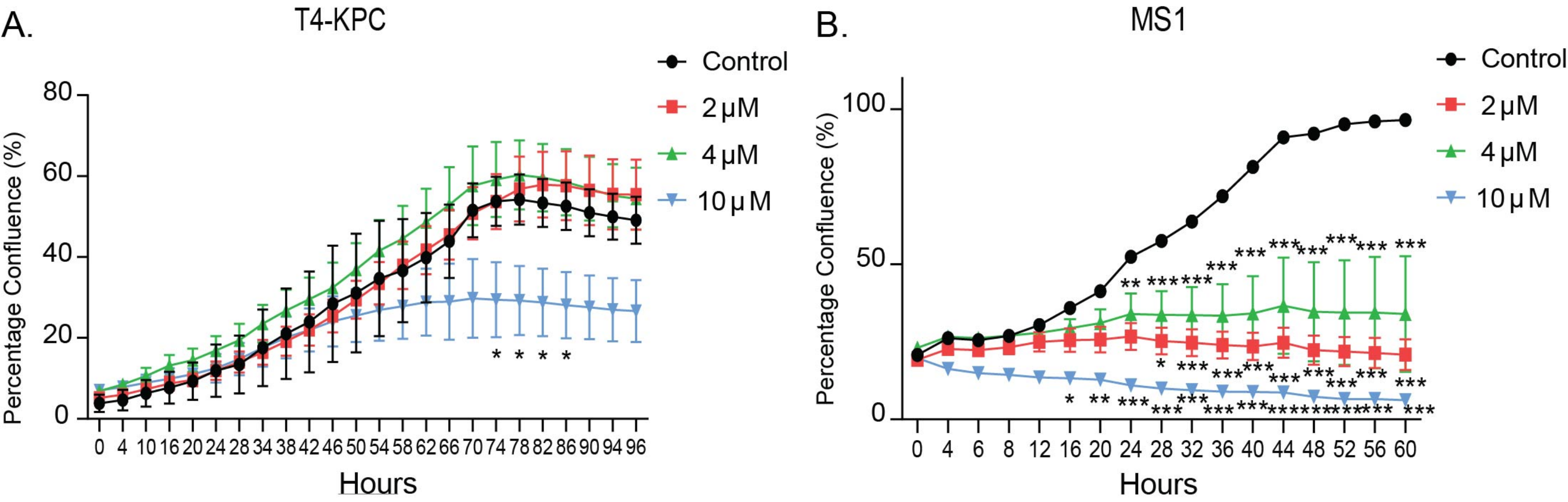

Supplementary Figure 5: Vps34 inhibition does not phenocopy 3-MA. A-B. Line graphs depicting cellular proliferation of T4-KPC and MS1 cells subjected to increasing doses of the Vps34 inhibitor VPS-IN1. Data is mean ± SEM compared with 2-way ANOVA with Bonferroni's test (A). *$p<0.05$; **$p<0.01$; ***$p<0.001$.

Supplementary Figure 6

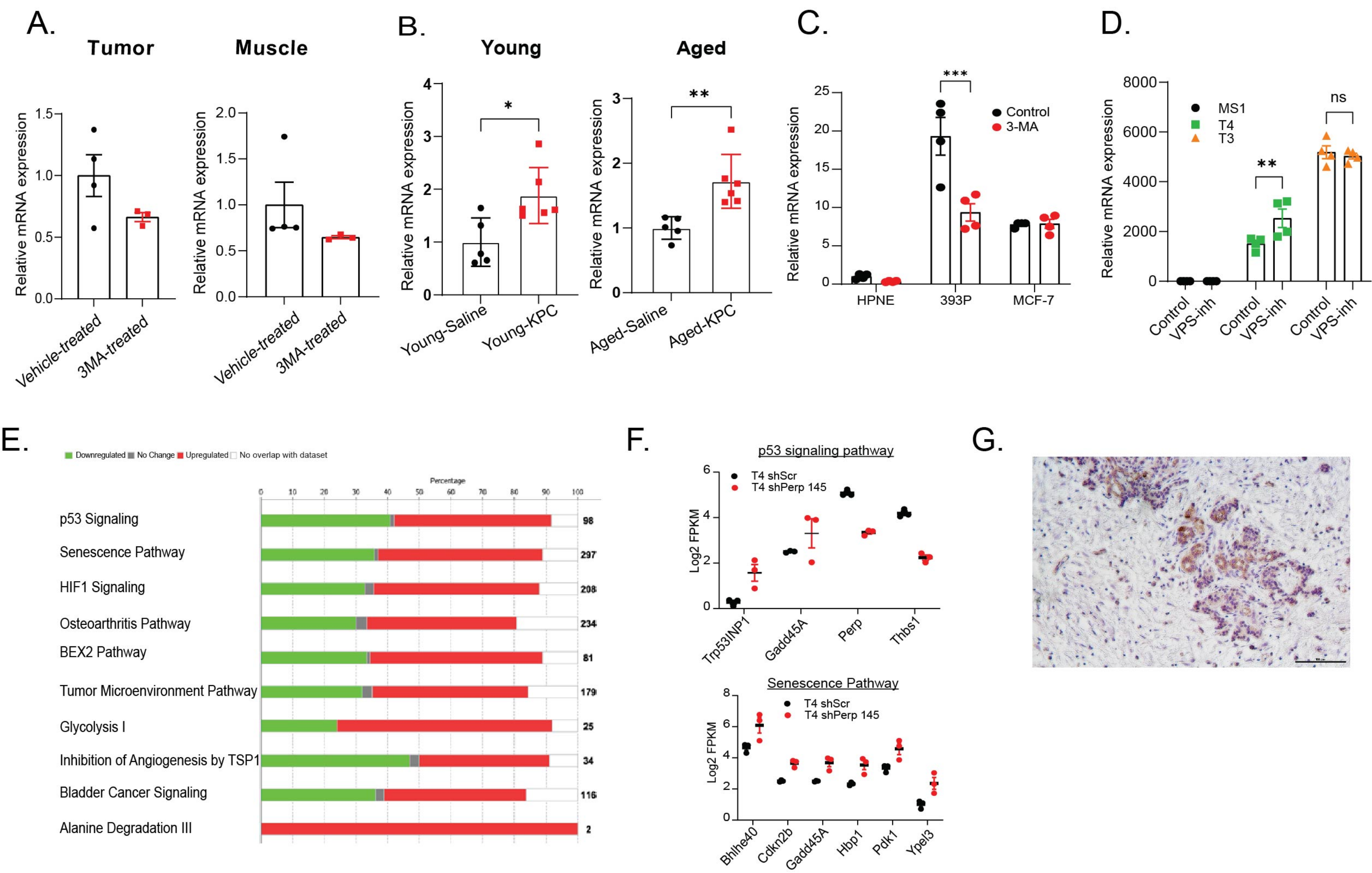

Supplementary Figure 6: *Perp* is elevated in mouse and patient tumors and is reduced by 3-MA. A. mRNA expression of *Perp* in the tumor (left) and muscle (right) of PDO mice with (n=3) and without (n=5) 3-MA treatment. B. mRNA expression of *Perp* in the muscles of young (left)/aged (right) control and tumor-bearing mice. C. mRNA expression of Perp in in HPNE, 393P and MC-7 +/- 3MA (5mM). D. mRNA expression of *Perp* +/- VPS-IN1 (10µM) for 24 hours. E. Top 10 significantly altered pathways in T4 shPerp 145 vs T4 shScr. F. Genes altered in the p53 and senescence pathways in T4shScr and T4 shPerp 145 G. PERP immunostaining in tumor-adjacent tissue in a patient. Scale bar is 100µm. Data is mean ± SEM compared with Student's t-test (A-B), with 2-way ANOVA with Bonferroni's test (C-D). *p<0.05; **p<0.01; ***p<0.001.

Supplementary Figure 7

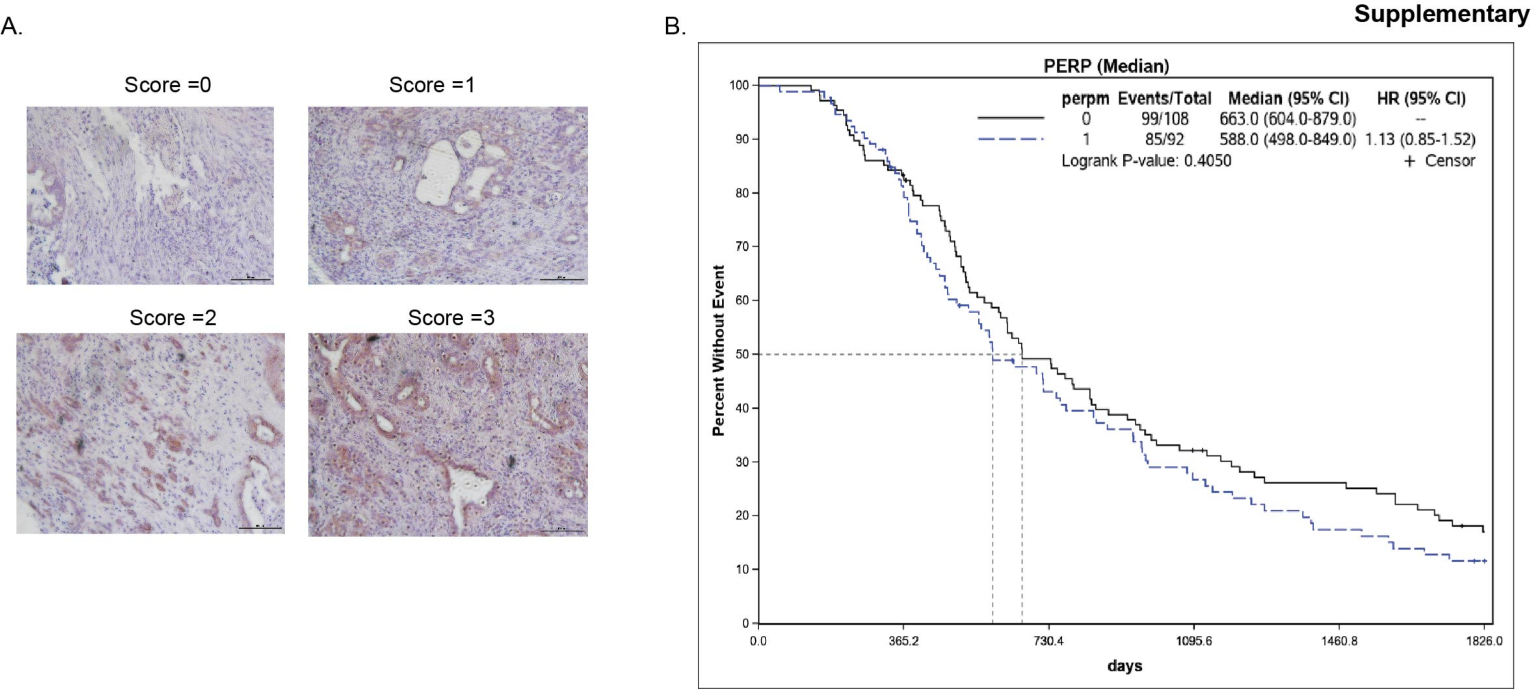

Supplementary Figure 7: Pancreatic cancer patients demonstrate increased staining of Perp in tumors. A. Histo-scores of tumor-microarray samples (n=200). Scale bar is 100μm. B. Survival analyses of patients having low and high-rank *Perp* expression.

Supplementary Figure 8

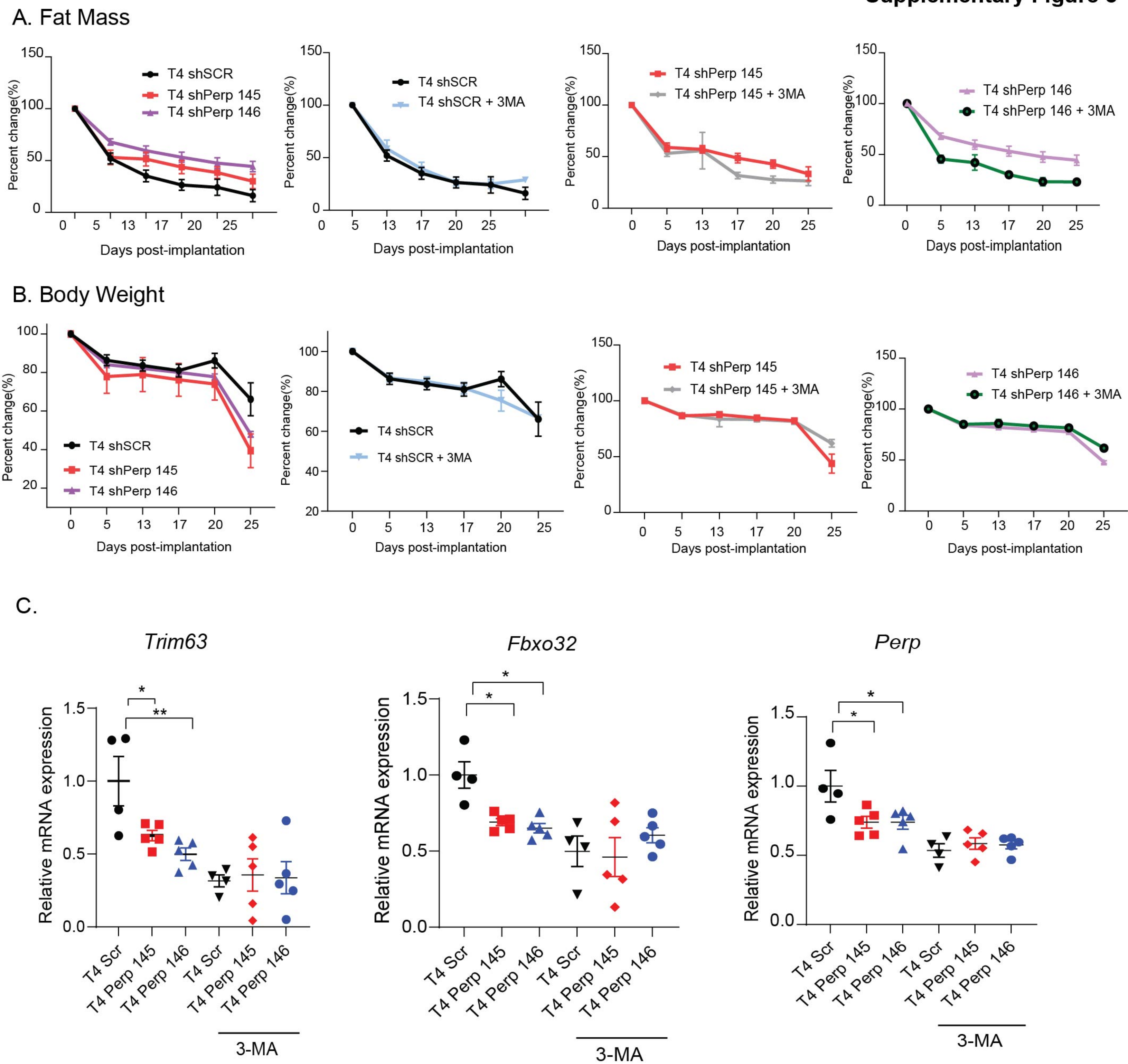

Supplementary Figure 8: Effect of 3MA on in vivo cachectic parameters. A. Fat Mass, B. Body Weight measurements C. mRNA expression of *Trim63*, *Fbxo32* and *Perp* of T4 shScr, T4 shPerp 145, T4 shPerp 146, T4 shScr +/- 3-MA, T4 shPerp 145 +/- 3-MA, T4 shPerp 146 +/- 3-MA. Data is mean ± SEM and compared using 2-way ANOVA with Bonferroni correction. *$p<0.05$; **$p<0.01$; ***$p<0.001$.
